# Landscape genetics reveals that adaptive genetic divergence in *Pinus bungeana* (Pinaceae) is driven by environmental variables relating to ecological habitats

**DOI:** 10.1186/s12862-019-1489-x

**Published:** 2019-08-01

**Authors:** Xue-Xia Zhang, Bao-Guo Liu, Yong Li, Ying Liu, Yan-Xia He, Zhi-Hao Qian, Jia-Xin Li

**Affiliations:** 1 Innovation Platform of Molecular Biology College of Forestry, Henan Agricultural University, No.95, Wenhua Road, Zhengzhou, 450002 China; 2 Guangdong Provincial Key Laboratory of Plant Resources, School of Life Sciences, Sun Yat-sen University, No.135, Xingang Xi Road, Guangzhou, 510275 China; 3 School of Life Sciences, Henan University, Kaifeng, 475004 China

**Keywords:** Adaptive genetic divergence, Adaptation potential, Environment-associated loci, Ecological niche modeling, *Pinus bungeana*, SCoT marker

## Abstract

**Background:**

Understanding the genetic basis of local adaptation has long been the concern of biologists. Identifying these adaptive genetic variabilities is crucial not only to improve our knowledge of the genetic mechanism of local adaptation but also to explore the adaptation potential of species.

**Results:**

Using 10 natural populations and 12 start codon targeted (SCoT) markers, a total of 430 unambiguous loci were yielded. The Bayesian analysis of population structure clearly demonstrated that the 10 populations of *P. bungeana* could be subdivided into three groups. Redundancy analysis showed that this genetic divergence was caused by divergence selection from environmental variables related to the ecological habitats of “avoidance of flooding” and “avoidance of high temperature and humidity.” LFMM results indicated that Bio1, Bio5, Bio8, Bio12, Bio14, and Bio16, which are related to the ecological habitat of *P. bungeana*, were correlated with the highest numbers of environment-associated loci (EAL).

**Conclusions:**

The results of EAL characterization in *P. bungeana* clearly supported the hypothesis that environmental variations related to the ecological habitat of species are the key drivers of species adaptive divergence. Moreover, a method to calculate the species landscape adaptation index and quantify the adaptation potential of species was proposed and verified using ecological niche modeling. This model could estimate climatically suitable areas of species spatial distribution. Taking the results together, this study improves the current understanding on the genetic basis of local adaptation.

**Electronic supplementary material:**

The online version of this article (10.1186/s12862-019-1489-x) contains supplementary material, which is available to authorized users.

## Background

Understanding the genetic basis of local adaptation is one of the core issues in the adaptive evolution of species and has long been the concern of biologists [1, 2]. Long-term local adaptation will force species to face two types of selection pressures from the environment: climatic fluctuations on a time scale and environmental heterogeneity on a spatial scale [3]. In response to these selection pressures, species will undergo adaptive genomic changes or plasticity and eventually yield phenotypic and phenological changes [4]. Accumulation of adaptive genomic changes contributes to the adaptive potential of a species. Identifying adaptive genetic variabilities can advance our understanding of the genetic mechanism of local adaptation and the adaptation potential of species [5]. However, determining these adaptive genetic variations in the genome responsible for local adaptation remains a great challenge for most nonmodel species because of the lack of available genomic information [6].

One of main goals of landscape genetics is to uncover the relationship between the adaptive genetic divergence in the genome and the environmental heterogeneity on a spatial scale [7]. Recent studies have made considerable progress in understanding species adaptive divergence in response to climatic fluctuations and environmental heterogeneity [8, 9]. In these studies, two major topics are addressed: (i) which environmental variables play key roles in the adaptive genetic divergence of a species and shape its landscape genetic structure, and (ii) which genes or loci on the genome undergo adaptive genetic differentiation [5]. Species adaptive genetic divergence and landscape genetic structure result from the combination of gene flow and natural selection [10]. Whereas natural selection from environmental variables often leads to adaptive differentiation of species genomes, gene flow can prevent the differentiation of most loci [11]. The end result is that the species genome produces genomic mosaics. Although adaptive differentiation among populations is influenced by natural selection yielded by the heterogeneous landscape, the landscape genetic structure of species is markedly affected by gene flow [12, 13]. Reduced gene flow due to geographical distance, disturbance from the heterogeneous landscape, barriers caused by geological or environmental factors, and dispersal mechanism of pollen and seeds of a species affects its landscape genetic structure. More studies are needed to confirm whether significant adaptive genetic divergence exists among populations determined by natural selection as influenced by environmental variables.

Previous studies have identified a multitude of adaptive genes or loci from a large number of species [14–17]. However, explaining why adaptive genetic changes occur at these genes is difficult. Current landscape genetic studies have proven that the adaptive genes or loci of species are usually diverse [18–20]. If species live in extreme environments, strong natural selection will force them to experience convergent genetic evolution [21, 22]. However, an extreme environment does not exist in most areas, but the adaptive differentiation of species living in these areas also varies. Recent landscape genetic studies put forward the hypothesis that environmental variations related to the ecological habitat of species are the key drivers of species adaptive divergence, that is to say, the genes related to these environmental variations will undergo adaptive differentiation [3, 22, 23]. Whether this hypothesis is also suitable for other species and has a certain universality remains unclear. Landscape genetic studies have paid considerable attention to the two topics mentioned above. Landscape genetics also provides an opportunity to assess species adaptive potential. Species adaptive potential is mainly affected by the ability to produce adaptive genes and transmit adaptive genes [8]. More adaptive gene production means a species can adapt to more complex climate fluctuations and heterogeneous landscapes, thus suggesting a stronger potential for adaptation [20, 24]. In addition, the adaptation potential of species depends on strong gene flow among populations, which may share adaptive genes and increase the adaptive capacity of neighboring populations [25]. Despite landscape genetic studies are beginning to focus on the adaptation potential of species [8, 25], developing a suitable way to measure this potential remains a problem. The population adaptive index, which is calculated using the percentage of adaptive loci with allele frequencies significantly different from those in other populations, has been previously reported [26]. However, the ability of adaptive genes to spread to other populations must be taken into account when considering the overall adaptability of the species. Herein, we devised a new index, i.e., the landscape adaptation index (LAI), to measure the adaptability of species.

*Pinus bungeana* Zucc. ex Endl. (Pinaceae), a coniferous evergreen tree that grows at 500–1800 m above sea level, is located in the warm temperate zone and subtropical margin of China [27]. This species is endemic to the country and has been listed as an endangered species by the IUCN. *P. bungeana* is a heliophilous plant; and it has a certain tolerance to cold and drought but is not resistant to flooding [28, 29]. The species grows well in cool and dry environments but thrives poorly at high temperatures and humidity. In this work, 10 natural populations of *P. bungeana* across its distribution area were used to investigate the adaptive divergence of the species by using the landscape genetic approach.

Start codon targeted (SCoT) polymorphism, a molecular marker with bias toward candidate functional genes, was developed based on short conserved sequences of start codons in plant genes [30]. This maker has the advantages of no requirement for genomic information, high repeatability, and high throughput [21]. In this study, we employed SCoT markers to scan the genome of *P. bungeana* and identify adaptive loci by analysis of associations with environmental data. The objectives of the study are to (i) examine the adaptive loci of *P. bungeana*, (ii) evaluate environmental variations associated with adaptive differentiation in *P. bungeana*, and (iii) establish a suitable LAI to measure the adaptation potential of species.

## Results

### Population genetic structure

**Fig. 1:**
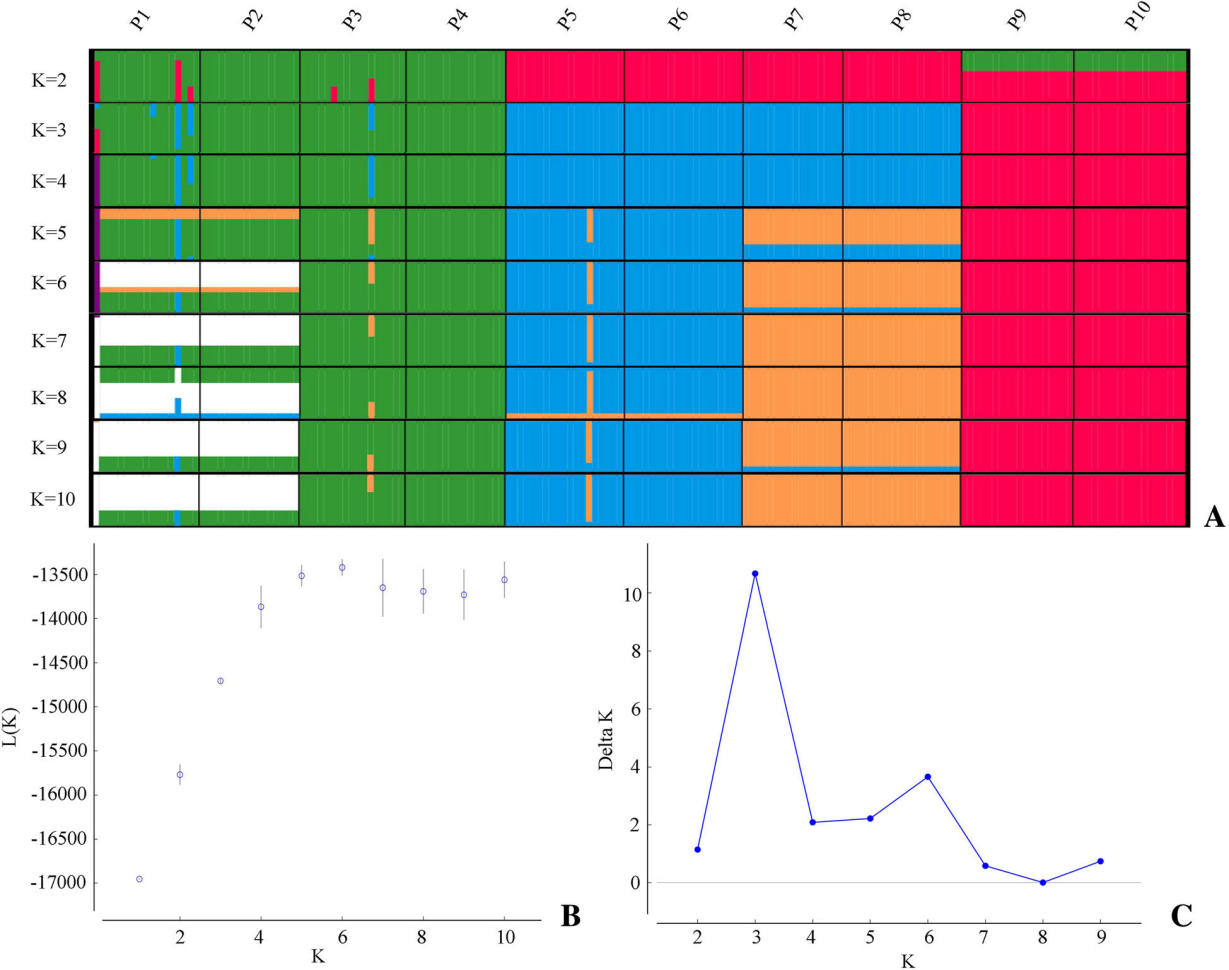
STRUCTURE analyses of ten sampled populations of *P. bungeana*. **a** Population genetic structure estimated by STRUCTURE analysis with K = 2 to K = 10. Each vertical bar shows the proportional representation of three population clusters (K) for an individual. **b** Values of log probability L(K) for the data as a function of K. **c**, Values of △K based on the second-order rate of change, with respect to K

A total of 175 individuals of *P. bungeana* from 10 natural populations were successfully scored using the 12 SCoT primers, and 430 unambiguous fragments were identified with sizes ranging from 60 bp to 1200 bp. The number of loci in 12 primers ranged from 17 (SCoT24) to 48 (SCoT25). The lowest number and percentage of polymorphic alleles (*N*_A_ = 87, *PPA* = 20.2) were found in the Gaopinghui, Sichuan (SCGP; P9) population and while the highest (*N*_A_ = 153, *PPA* = 35.6) were found in the Nannao Mt., Shanxi (SXNN; P1) population. The level of Nei’s genetic diversity (*H*_E_) of each population ranged from 0.069 in Lijiazhai, Hubei (HBLJ; P10) to 0.143 in SXNN (P1). Overall summary statistics of the genetic diversity analyses of 10 populations of *P. bungeana* are presented in Table 1. Bayesian analysis of the population structure of *P. bungeana* (Fig. 1) clearly demonstrated that the highest *ΔK* value is obtained when the 10 populations are subdivided into three groups. The first group is the middle group (P1 − P4), the second group is the north group (P5 − P8), and the third group is the south group (P9 − P10). Despite the significant genetic subdivision observed in the 10 populations of *P. bungeana*, only a small percentage of the genetic variation existed among the three subgroups (21.88%, *F*_CT_ = 0.219, *P* < 0.001; Table 2), and most of the genetic variation found within populations (63.08%, *F*_ST_ = 0.369, *P* < 0.001; Table 2).

**Table 1:** Details of population locations, sample size, genetic diversity of ten populations for *P. bungeana*

Population no. and code	Locations	Altitude (meters)	Lat.(N)/ Long.(E)	*N*	*N*_A_	*PPA*	*H*_E_
Middle group							
1.SXNN	Nannao Mt., Shanxi	955	36.33/113.51	17	153	35.6	0.143
2.GSLG	Lingguan Mt., Gansu	1220	33.94/106.36	16	96	22.3	0.077
3.SXLJ	Laojun Mt., Shaanxi	1436	34.33/110.19	17	109	25.3	0.095
4.SXWZ	Wuzi Mt., Shaanxi	748	32.95/107.85	16	100	23.3	0.077
North group							
5.SXWJ	Wujin Mt., Shanxi	1198	37.86/112.77	19	129	30	0.104
6.HNSN	Shennong Mt., Henan	1028	35.22/112.80	19	124	28.8	0.094
7.SXWL	Wulao Mt., Shanxi	1355	34.81/110.58	16	100	23.3	0.083
8.GSMJ	Maiji Mt., Gansu	1572	34.35/106.01	19	97	22.6	0.085
South group							
9.SCGP	Gaopinghui, Sichuan	1091	32.23/105.07	18	87	20.2	0.071
10.HBLJ	Lijiazhai, Hubei	712	31.90/111.61	18	91	21.2	0.069

*N*_A_ number of polymorphic alleles, *PPA* percentage of polymorphic alleles, *H*_E_ Nei’s (1973) measure of gene diversity

**Table 2:** Hierarchical AMOVAs for SCoT variation surveyed in *P. bungeana*

Source of variation	d.f.	%Total variance	*F*-statistic	*P*-value
Among three groups	2	21.88	*F*_CT_ = 0.219	*P* < 0.001
Among populations	7	15.05	*F*_SC_ = 0.193	*P* < 0.001
Within populations	165	63.08	*F*_ST_ = 0.369	*P* < 0.001

**Fig. 2:**
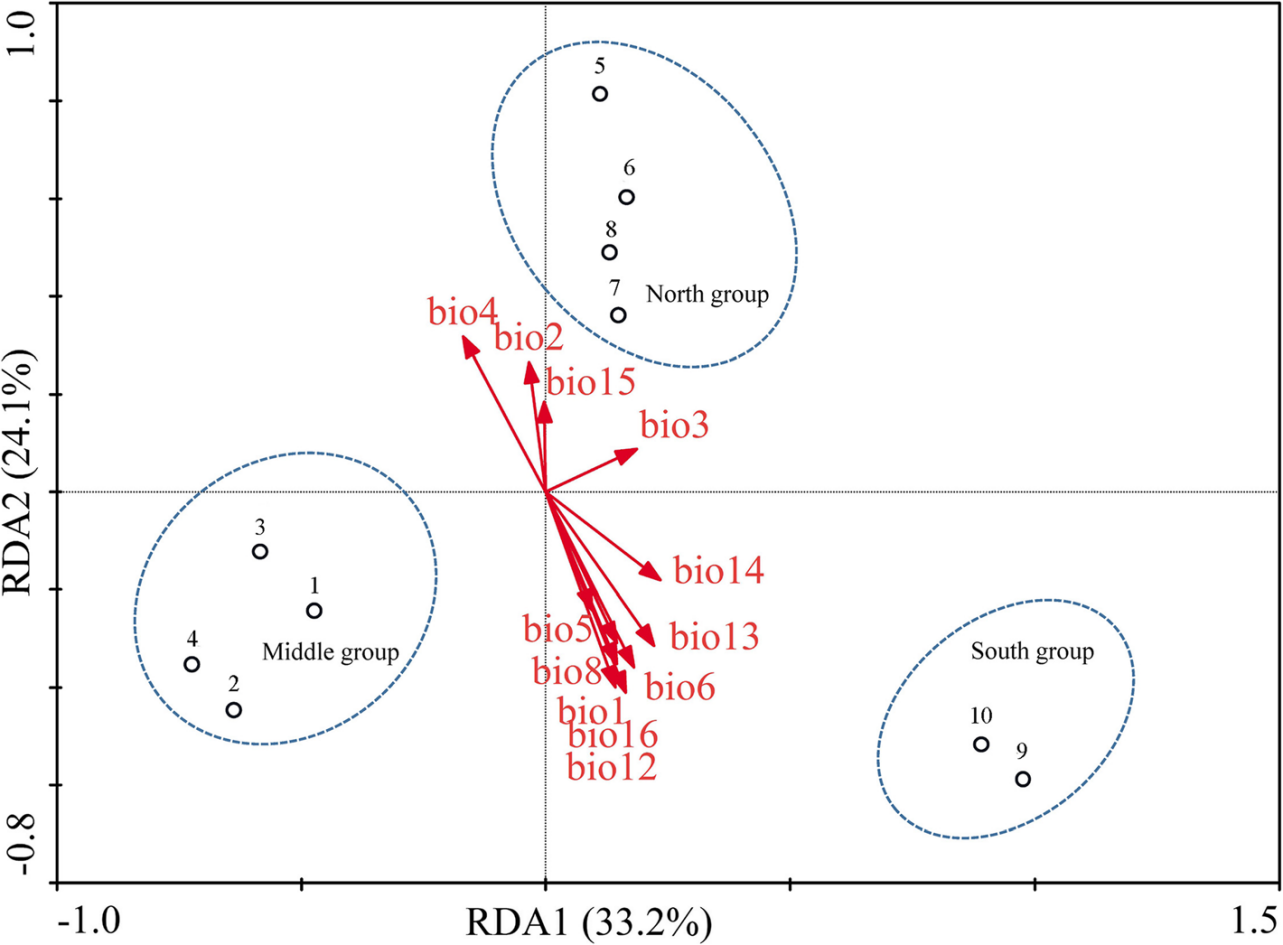
RDA analysis of *P. bungeana* showing the relative contribution of each environmental variation shaping population genetic structure. The biplot depicts the eigenvalues and lengths of eigenvectors for the RDA

Redundancy analysis (RDA) was performed to detect the roles of several environmental variables in this genetic subdivision, as well as the relative contribution of each variable to the population genetic structure, and the results are presented in Fig. 2 and Table 3. The associations of gene frequencies of 430 alleles with 12 environmental variables in axes 1 and 2 were both 1.000, and the percent variances of relation between genetic and environmental variables in axes 1 and 2 were 33.2 and 24.1%, respectively. RDA revealed that these environmental variables significantly divide *P. bungeana* into three groups, consistent with the results of STRUCTURE analysis. Seven environmental variables were significantly correlated with RDA axis 2 (Table 3). Among these variables, annual mean temperature (Bio1), temperature seasonality (Bio4), minimum temperature of the coldest month (Bio6), and mean temperature of the wettest quarter (Bio8) are temperature variables, while annual precipitation (Bio12), precipitation of the wettest month (Bio13), and precipitation of the wettest quarter (Bio16) are precipitation variables. Bio12 and Bio16 showed the strongest correlations among the seven environmental variables.

**Table 3:** Correlations between environmental variables and the ordination axes

Environmental variable	Axis 1	Axis 2	Axis 3	Axis 4
Bio1	0.313	−0.731*	0.006	0.353
Bio2	−0.059	0.542	−0.581	−0.211
Bio3	0.323	0.178	−0.729*	−0.143
Bio4	−0.294	0.648*	−0.259	−0.144
Bio5	0.164	−0.494	−0.412	0.305
Bio6	0.252	−0.717*	0.222	0.286
Bio8	0.252	−0.629*	−0.075	0.406
Bio12	0.247	−0.814**	0.184	0.271
Bio13	0.385	−0.640*	−0.261	0.244
Bio14	0.406	−0.369	0.153	0.049
Bio15	−0.006	0.376	−0.566	−0.154
Bio16	0.284	−0.836**	−0.037	0.227

Statistically significant correlation by * (*P* < 0.05) and ** (*P* < 0.01)

### Characterization of environment-associated loci (EAL)

**Fig. 3:**
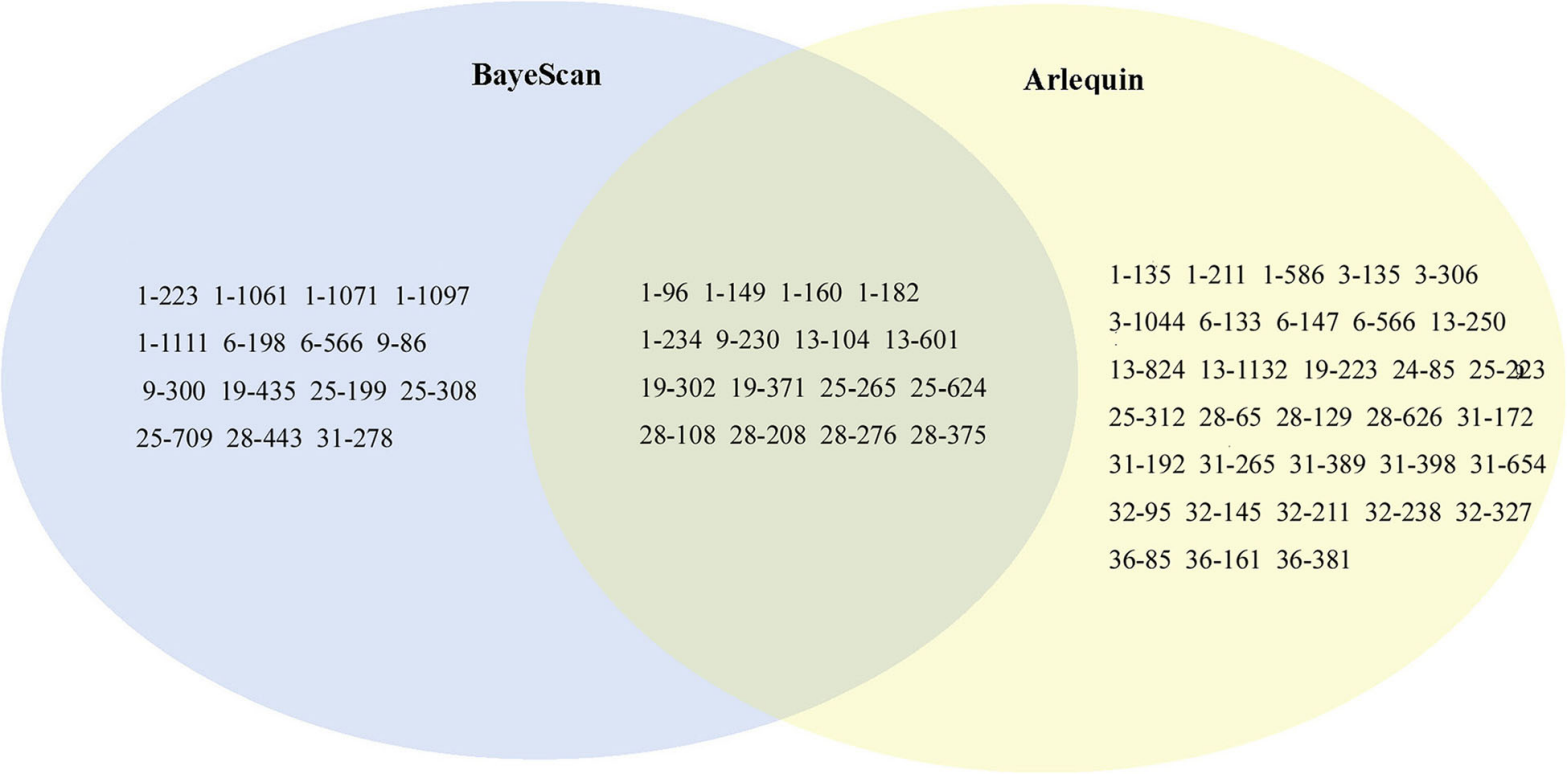
The results of outlier loci detected by Bayescan and Arlequin. Sixty-seven, 49, and 20 loci were identified as outlier loci in *P. bungeana* using Bayescan, Arlequin, and both with Bayescan and Arlequin, respectively

Using the hierarchical island model in Arlequin, a total of 49 outlier loci (11.4% of 430 SCoT loci) above the 95% threshold were identified (Fig. 3; Online Resource 4). Using the Bayesian method in BayeScan, a total of 31 outlier loci (7.2% of 430 SCoT loci) with posterior probability above 0.76 (i.e., log10 PO > 0.5) were identified (Fig. 3; Additional file 1), representing a threshold of substantial evidence under selection. Because of the characteristics of the two identification methods, we sought a common result to reduce the false discovery rate. Here, a total of 16 mutual loci (3.7% of 430 SCoT loci), representing robust outlier loci (Fig. 3), were identified by both methods. Latent factor-mixed modeling was performed in software LFMM to verify which of these loci are driven by environmental variables, and 13 EAL (3.0% of 430 SCoT loci) associated with at least one environmental variable were identified (Table 4). Linkage disequilibrium analysis showed these EAL are all unlinked loci and significantly correlated with both temperature and precipitation (Table 4). Among the 12 environmental variables found, Bio1, Bio5, Bio8, Bio12, Bio14, and Bio16 were correlated with the highest numbers of EAL. This result shows that these environmental variables play key roles in the adaptive divergence of *P. bungeana* (Table 4).

**Table 4:** The EAL as indicated by |z|-score

Locus	Bio1	Bio2	Bio3	Bio4	Bio5	Bio6	Bio8	Bio12	Bio13	Bio14	Bio15	Bio16
1–96	7.551^***^	3.413^***^		6.455^***^	4.263^***^	6.613^***^	6.262^***^	8.017^***^	9.238^***^	5.772^***^		9.145^***^
1–149	8.047^***^	4.008^***^		7.176^***^	4.368^***^	7.369^***^	6.592^***^	8.046^***^	8.593^***^	5.463^***^		9.005^***^
1–160	8.873^***^	5.400^***^		8.835^***^	4.021^***^	9.008^***^	6.719^***^	9.785^***^	8.538^***^	5.036^***^		10.311^***^
1–182	8.475^***^	3.509 ^***^		7.030^***^	5.088^***^	7.278^***^	7.016^***^	8.377^***^	9.607^***^	5.732^***^		9.688^***^
1–234	8.455^***^	3.295^***^		6.850^***^	5.276^***^	7.144^***^	7.105^***^	8.342^***^	9.842^***^	5.739^***^		9.654^***^
13–104	5.457^***^	3.826^***^		3.300^***^	7.346^***^	5.276^***^	6.277^***^	6.459^***^			4.053^***^	5.847^***^
19–302	8.185^***^			6.141^***^	5.468^***^	6.738^***^	7.184^***^	7.981^***^	8.945^***^	6.389^***^		8.764^***^
19–371	7.853^***^			5.787^***^	5.610^***^	6.376^***^	6.997^***^	7.768^***^	9.619^***^	6.426^***^		8.811^***^
25–265	3.753^***^	7.734^***^	3.344^***^	8.371^***^		6.403^***^		5.490^***^		5.567^***^	5.449^***^	3.653^***^
25–624		4.069^***^	6.446^***^								3.461^***^	
28–108	8.677^***^	3.386^***^		6.559^***^	5.652^***^	7.219^***^	7.711^***^	8.641^***^	9.872^***^	6.750^***^		9.427^***^
28–208	3.911^***^	3.332^***^	8.849^***^		6.821^***^		5.090^***^		8.208^***^	4.304^***^		3.651^***^
28–375	4.828^***^		5.886^***^		5.695^***^		5.247^***^	3.406^***^	7.548^***^	4.336^***^		4.846^***^

Statistically significant correlation by ^***^ (*P* < 0.001)

### Adaptation potential of *P. bungeana*

**Fig. 4:**
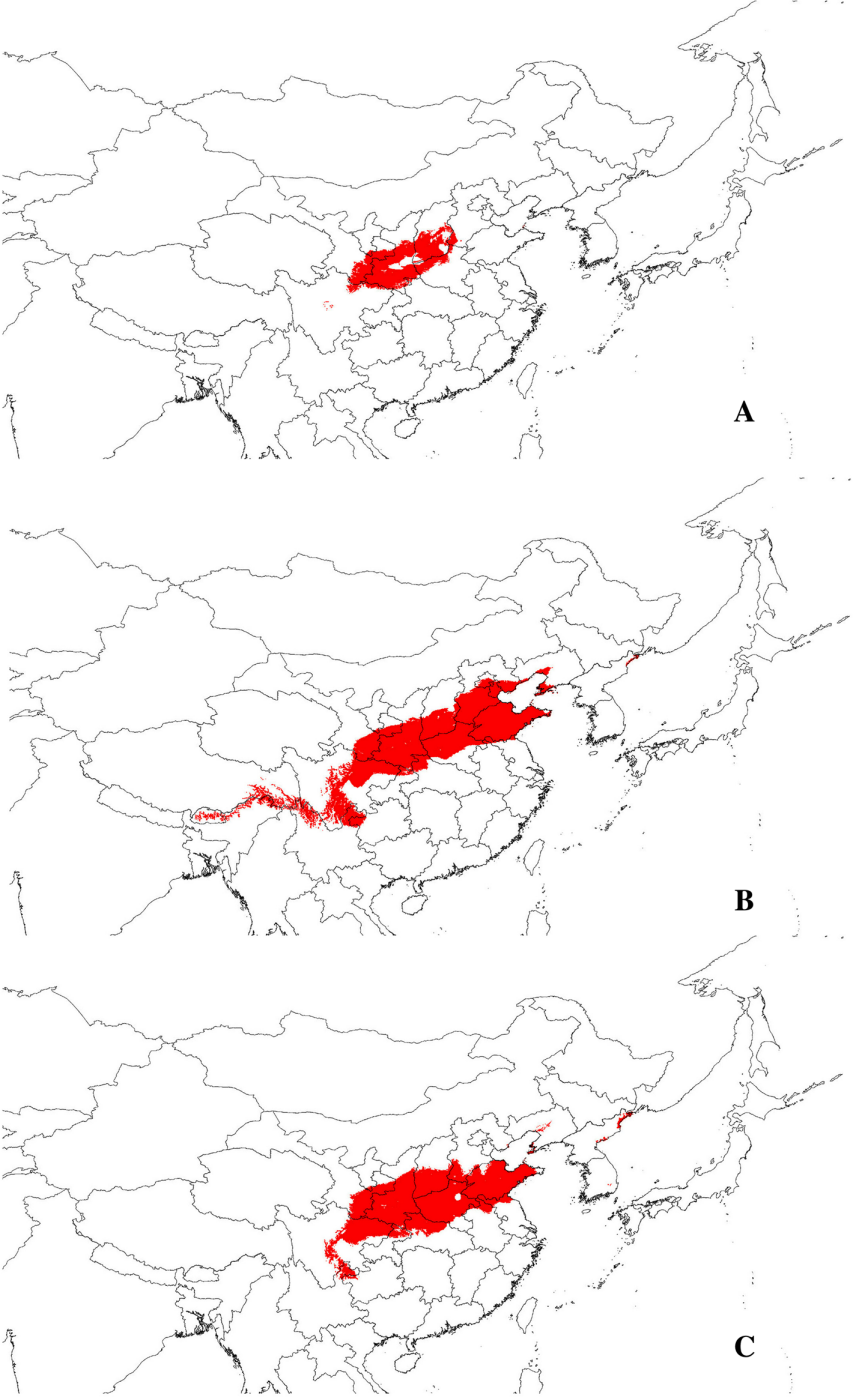
Maps showing the bioclimatic suitability (logistic value > 0.50) through ecological niche modeling with Maxent using bioclimatic variables. Map yielded by software DIVA-GIS, the software and free spatial data were downloaded from http://www.diva-gis.org/download/. **a** for *P. bungeana*; **b** for *C. coggygria*; **c** for *F. suspensa*

Based on the LAI calculation method introduced in Methods, the LAI of *P. bungeana* was found to be 0.607. To verify this index, we also calculated the LAIs of two other species in the same region, namely, *Cotinus coggygria* and *Forsythia suspensa*, for comparison. The LAIs of *C. coggygria* and *F. suspensa* were 1.249 and 1.005, respectively. To investigate the adaptability of the these species, their climatically suitable areas were reconstructed using Maxent. The areas predicted to be climatically suitable for *P. bungeana*, *C. coggygria*, and *F. suspensa* are illustrated in Fig. 4. Climatically suitable areas with logistic values above 0.5 for *P. bungeana* (318,775 km^2^) were significantly smaller than those for *C. coggygria* (1,013,266 km^2^) and *F. suspensa* (839,363 km^2^). This result could be matched to the LAIs calculated earlier.

## Discussion

Our study investigated the spatial distribution of genetic variations in natural populations of *P. bungeana* across its entire distribution range to provide insights into the complex interaction between environment variables and the adaptive genetic divergence of this conifer species. This work raises our understanding of the interaction of evolutionary processes, such as natural selection, gene flow, and local adaptation. Landscape genetics research has proven to be an effective approach to reveal this interaction [4, 5]. In the present study, 430 SCoT loci were used to obtain insights into the potential adaptive divergence of *P. bungeana* in response to a heterogeneous landscape.

Revealing the role of environmental variables in the formation of the spatial population genetic structure of species is a major concern of landscape genetics [31, 32]. Although such studies have begun to gain popularity in landscape genetic research, accurately determining the role of environmental variables in the spatial population genetic structure remains challenging because the spatial genetic structure of species is often shaped by many factors, such as natural selection, gene flow, genetic drift, geographical distance, ecological distance, geographical isolation, ecological isolation, geologic events, and population demographic history [3, 33]. Our results showed the significant genetic divergence of natural populations of *P. bungeana*. Ten populations from *P. bungeana* are significantly clustered into three genetic groups, namely, the north group (P5 − P8), the middle group (P1 − P4), and the south group (P9 − P10).

Three perspectives may explain the significant genetic division of species observed. First, genetic division may be caused by past population dynamics. The current distribution pattern of *P. bungeana* is the result of redistribution from three glacial refugia during Quaternary glaciation. Second, genetic division may occur due to environmental or geographical isolation. Geographical or environmental barriers existent among the three groups may hinder gene flow and lead to significant genetic differentiation. Finally, genetic division may arise from natural selection. Given significant differences in the habitat environments of the species considered, the selection pressure caused by such heterogeneity could result in the divergent selection of species, leading to significant divergence of the three groups. Assuming that the first explanation is true, populations with the highest genetic diversity can be considered as glacial refugia, whereas populations located far from the refugia can show a gradually reduction in genetic diversity due to the founder effect. The results of the middle group (P1 − P4) support this hypothesis, but those of the north group (P5 − P8) clearly do not meet expectations. We do not consider the results of the south group because it only has two populations. Furthermore, previous phylogeographic studies of *P. bungeana* have confirmed that it has only one or two refugia and that these refugia are located in the western areas of the Qinling-Daba and Lvliang Mountains [34, 35]. The results of these studies clearly do not support our first hypothesis. Overall, the first explanation cannot appropriately explain the genetic divergence of *P. bungeana*. By assuming that genetic divergence of *P. bungeana* is caused by geographical or environmental barriers, we would expect to find significant genetic differences among these three groups. The results of hierarchical AMOVA (*F*_CT_ = 0.219, *P* < 0.001) seem to support this hypothesis. In fact, the distribution of populations from the three groups of *P. bungeana* is continuous, especially for the north and middle groups. No obvious geographic barrier, i.e., mountains or rivers, separates the three groups. Thus, we may consider an alternative explanation: the genetic divergence among the three groups of *P. bungeana* is caused by environmental barriers. However, according to the results of our simulation of the climatically suitable areas of *P. bungeana*, these climatically suitable areas are distributed continuously, and no ecological isolation among the three groups could be observed (Fig. 4a). Hence, the second explanation cannot be used to explain the genetic divergence of the three groups of *P. bungeana*. In the third perspective, reductions in the magnitude of gene flow could be expected because excessively strong gene flow will blur the population genetic differentiation caused by a heterogeneous environment. The value of gene flow (*N*_*m*_ = 0.809) based on neutral loci was consistent with this expectation. RDA was performed to verify whether the genetic divergence of the three groups of *P. bungeana* is caused by divergence selection from heterogeneous environments. The RDA results demonstrated that the 12 environmental variables unambiguously divided 10 populations of *P. bungeana* into the south group, the middle group, and the north group (Fig. 3). Among the 12 environmental variables, Bio12 and Bio16, which refer to annual precipitation and precipitation of the wettest quarter, respectively, were the most important environmental factors shaping the genetic divergence of the three groups of *P. bungeana*. The results suggest that precipitation, especially precipitation of the wettest quarter, exerts very strong selection pressure on *P. bungeana.* The times of the wettest and warmest seasons in this region notably overlap. This finding is also consistent with the ecological habitats of “avoidance of flooding” and “avoidance of high temperature and humidity.” Overall, our results for *P. bungeana* support the third explanation of genetic division.

Another concern in landscape genetic studies involves the identification of adaptive loci [4, 36]. Although adaptive gene loci and their environmental drivers have been identified in landscape genetic studies in recent years [2, 9], little attention has been paid to why they are these loci or drivers. Recent landscape genetic studies hypothesize that environmental variations related to the ecological habitats of species are the key drivers of species potential adaptive divergence and that the related genes undergo adaptive differentiation [3, 22, 23]. Whether this hypothesis is universal has yet to be verified by more landscape genetic studies. In the present study, we selected *P. bungeana*, an important coniferous evergreen tree, as a model to examine this concern. The results of LFMM showed that Bio1, Bio5, Bio8, Bio12, Bio14, and Bio16 are correlated with the highest numbers of EAL. Among these six environmental variables, Bio1, Bio5, and Bio8, which refer to annual mean temperature, max temperature of warmest month, and mean temperature of wettest quarter, respectively, are temperature-relevant variables, while Bio12, Bio14, and Bio16, which refer to annual precipitation, precipitation of driest month, and precipitation of the wettest quarter, respectively, are precipitation-relevant variables. Bio1, Bio5, and Bio8 are associated with the ecological habitats of “preference of cool” and “avoidance of high temperature and humidity,” while Bio12, Bio14, and Bio16 are associated with the ecological habitats of “tolerance of cold and drought,” “avoidance of flooding,” and “avoidance of high temperature and humidity.” Thus, the above hypothesis on the role of environmental variations is also suitable for *P. bungeana*.

Moving beyond identification of adaptive genetic variation, landscape genetics provides researchers an opportunity to assess species adaptive potential. The adaptation potential of species depends mainly on two factors: the ability to produce adaptive genes and the ability to spread these genes out [8]. Thus, we devised a new index, i.e., LAI, to measure the adaptation potential of species. The LAIs of *P. bungeana*, *C. coggygria*, and *F. suspensa* were estimated according to our calculation method, and results showed that *C. coggygria* (LAI = 1.249) has the highest adaptive ability among the three species whereas *P. bungeana* (LAI = 0.607) has the lowest. This finding raises an important question of whether the index is reasonable. Thus, we sought a valid indicator to test this index. The adaptation potential of species is mainly manifested in two aspects: the ability of species to cope with climate change and the ability of species to distribute in space. As predicting and quantifying the ability of species to adapt to climate change is difficult, we used ecological niche modeling to calculate climatically suitable areas to represent the ability of spatial distribution of species. The results showed that *C. coggygria* (1,013,266 km^2^) has the largest climatically suitable areas and that the climatically suitable areas of *F. suspensa* (839,363 km^2^) and *P. bungeana* (318,775 km^2^) are smaller than that of *C. coggygria*. This result clearly supports the LAIs of these three species. Although previous studies have suggested that climate change and human disturbance are the main reasons for the endangerment of *P. bungeana* [29], our results of low LAI show that low adaptive differentiation ability is also one of the reasons for its endangerment. Therefore, reducing human disturbance and preventing rapid climate change may be the most effective strategies to protect the natural population of *P. bungeana*.

Despite its benefits, a number of problems related to our LAI that may lead to an underestimation of species adaptation potential must be noted. For instance, we only considered the current status of differentiation of adaptive genes, which might produce more differentiated genotypes in the past. In addition, increases in human activities in the warm temperate and subtropical regions of China could seriously interfere with gene flow among species populations [37, 38]. The ability to transfer genes based on the current gene flow may be underestimated. Overall, the LAI we proposed is useful for quantifying species adaptability.

## Conclusions

In this study, SCoT markers were adopted to investigate the adaptive genetic divergence of *P. bungeana.* Our results showed that environmental variables related to ecological habitat play a key role in the adaptive genetic divergence of species. We also proposed a method to calculate the LAI of a species and quantify its adaptation potential and then verified this index by using ecological niche modeling to estimate the climatically suitable areas of species spatial distribution. Our results showed that *P. bungeana* has a low LAI, which suggests that low adaptive differentiation ability is also responsible for its endangerment. We believe that, although it requires a larger number of species for verification, the LAI proposed in this work will enhance the current understanding of the adaptation potential of various species.

## Methods

### Sample collection

**Fig. 5:**
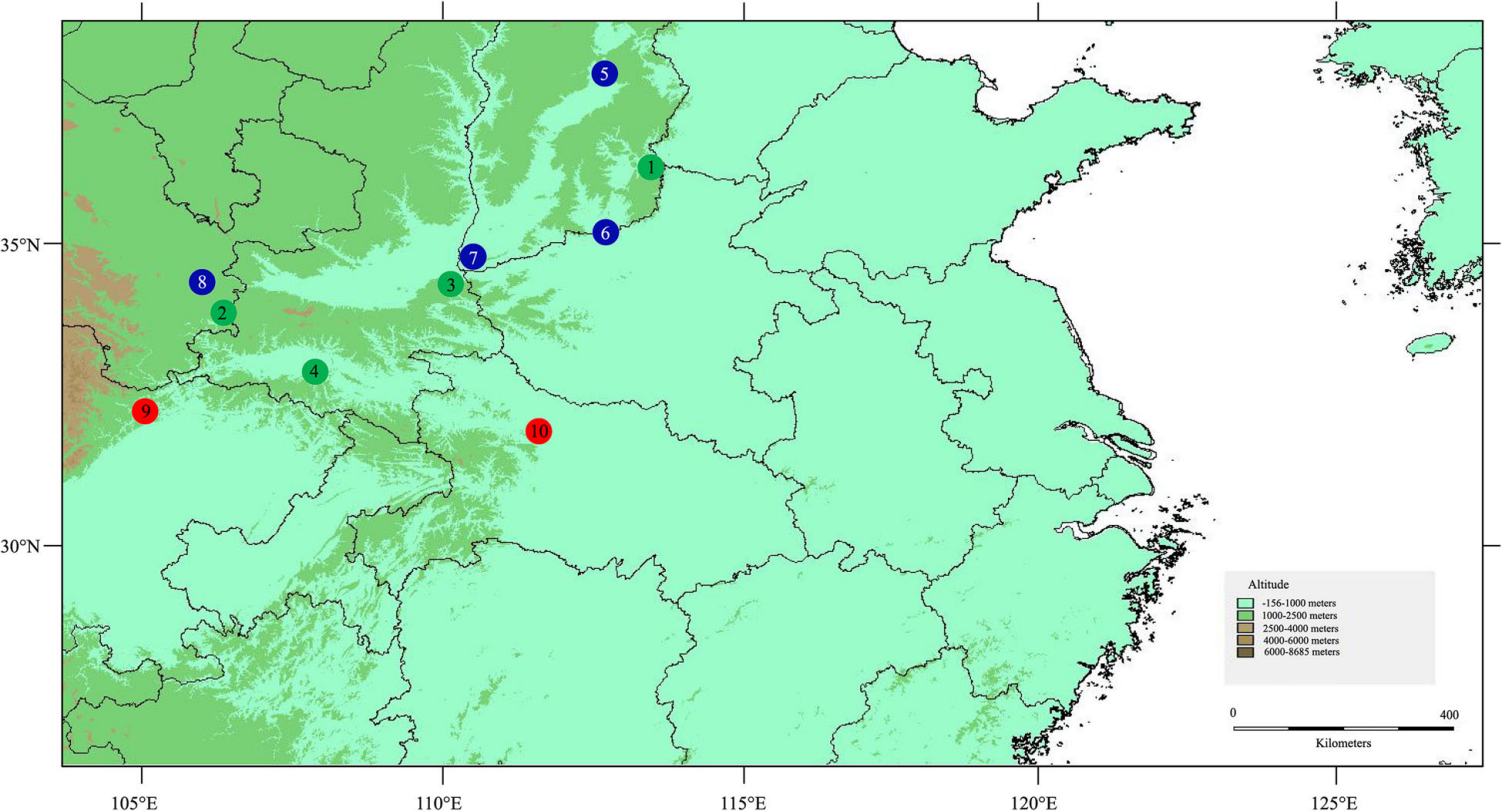
Locations of the ten sampled *P. bungeana* populations. Map yielded by software DIVA-GIS, the software and free spatial data were downloaded from http://www.diva-gis.org/download/

A total of 175 individuals from 10 natural populations were collected across the entire distribution range of *P. bungeana* in China (Fig. 5). Population samples contained 16–19 individuals (Table 1), and each individual was collected at least 20 m apart. Young, healthy needles were sampled and stored in a silica gel in zip-lock bags at room temperature until DNA extraction. The geographical information and numbers of individuals for each sampled population are presented in Table 1. After identified by Dr. Yong Li, voucher specimens (voucher no. LiPb2017001–2,017,010) were deposited at the herbarium of College of Forestry, Henan Agricultural University. No specific permissions were required for *P. bungeana*, the collection of plant material complied with current Chinese regulations.

### Molecular protocols

We used the methodology previously described by Yang et al. [3]. DNA was extracted from approximately 30 mg of dried needles by using plant DNA extraction kits (Tiangen, Beijing, China). DNA was quantified with an ND5000 microvolume spectrophotometer (BioTeke, Beijing, China). All individuals were genotyped with SCoT markers. Despite limitations inherent in the lack of DNA sequence information, SCoT loci are suitable for landscape genetic studies due to their wide distribution across the genome and ability to target functional regions [3]. Four individuals from different populations were randomly selected to verify their polymorphism and repeatability. After an initial screening, the 12 best primers (i.e., SCoT1, SCoT3, SCoT6, SCoT9, SCoT13, SCoT19, SCoT24, SCoT25, SCoT28, SCoT31, SCoT32, and SCoT36) were retained for polymerase chain reaction (PCR). SCoT1, SCoT13, SCoT25, and SCoT32 were 5′ fluorescent primers labeled with FAM; SCoT3, SCoT9, SCoT19, and SCoT24 were labeled with HEX; and SCoT6, SCoT28, SCoT31, and SCoT36 were labeled with TAMRA. The reaction mixtures and conditions of PCR amplification was descriped in Yang et al. [3]. The primer-specific annealing temperatures were 50 °C for SCoT19; 54 °C for SCoT6 and SCoT9; 56 °C for SCoT1, SCoT13, SCoT24, SCoT25, SCoT28, SCoT31, SCoT32, and SCoT36; and 60 °C for SCoT3. Finally, 3 μL of PCR products was mixed with 10 μL of HiDi formamide and ran on an ABI 3730 DNA analyzer at BGI (Beijing, China). PCR products were measured with the internal size standard of Liz1200 (Applied Biosystems, Foster City, USA).

### Data analysis

The SCoT raw data were scored and transformed into a 1/0 matrix according to the presence or absence of peaks visualized using GeneMarker 2.2.0 software (SoftGenetics, State College, Pennsylvania, USA). To minimize false scoring, peaks within 60–1200 bp and relative fluorescent units above 500 were scored. The following population genetic analyses were conducted on the basis of the 1/0 matrix of SCoT markers.

Genetic parameters, including polymorphic allele number (*N*_A_), Nei’s measure of gene diversity (*H*_E_) [39], percentage of polymorphic alleles (*PPA*), and gene frequencies per allele were estimated using AFLPSURV 1.0 [40]. Analysis of the population genetic structure of the 10 populations of *P. bungeana* was implemented using the Bayesian-based program STRUCTURE 2.3.4 [41]. STRUCTURE was run with *K* values from 1 to 10 using the no-admixture model with independent allele frequencies. Ten replicate runs of 100,000 Markov chain Monte Carlo iterations and a burn-in period of 100,000 repetitions were performed for each value of *K*. The optimal *K* that best represented the major population structure was assessed according to the method introduced by Evanno et al. [42], which was performed using Structure Harvester [43]. CLUMPP 1.1 was used to calculate the average estimated admixture coefficients obtained over the 10 runs [44]. Histograms of the STRUCTURE results were produced using DISTRUCT 1.1 [45], and hierarchical analysis of molecular variance (AMOVA) was carried out in ARLEQUIN 3.5 [46] to infer the distribution of genetic differentiation at various levels. Nineteen environmental variables of Worldclim (http://www.diva-gis.org/climate) from 1950 to 2000 at 2.5 arcmin resolution were downloaded and extracted using DIVA-GIS 7.5 [47]. In this study, uncorrelated environmental variables were used for subsequent RDA and environmental association analyses. Pearson correlation analysis was performed in SPSS 19 (SPSS Inc., Chicago, IL, USA) to eliminate strongly correlated environmental variables with a correlation value higher than 0.95 [22]. Twelve uncorrelated environmental variables (i.e., Bio1, Bio2, Bio3, Bio4, Bio5, Bio6, Bio8, Bio12, Bio13, Bio14, Bio15, and Bio16) were retained. To identify the contribution of environmental variables to the population genetic structure, RDA, a constrained linear ordination method, was performed in CANOCO 4.5 [48]. Here, we used gene frequencies per allele in each population (Additional file 2) as the response variable and the 12 uncorrelated environmental variables (Table 5 and Additional file 3) as explanatory variables.

**Table 5:** Nineteen environmental variables used in this study

Temperature (period 1950–2000)	Bio1: Annual mean temperature (°C)
	Bio2: Mean diurnal range (Mean of monthly (max temp - min temp))
	Bio3: Isothermality (Bio2/Bio7) (×100)
	Bio4: Temperature seasonality (standard deviation ×100)
	Bio5: Max temperature of warmest month (°C)
	Bio6: Min temperature of coldest month (°C)
	Bio7: Temperature annual range (E5-E6)
	Bio8: Mean temperature of wettest quarter (°C)
	Bio9: Mean temperature of driest quarter (°C)
	Bio10: Mean temperature of warmest quarter (°C)
	Bio11: Mean temperature of coldest quarter (°C)
Precipitation (period 1950–2000)	Bio12: Annual precipitation (mm)
	Bio13: Precipitation of wettest month (mm)
	Bio14: Precipitation of driest month (mm)
	Bio15: Precipitation seasonality (coefficient of variation)
	Bio16: Precipitation of wettest quarter (mm)
	Bio17: Precipitation of driest quarter (mm)
	Bio18: Precipitation of warmest quarter (mm)
	Bio19: Precipitation of coldest quarter (mm)

Two approaches were used to identify outlier loci and reduce the false discovery rate. In the first approach, the hierarchical island model in Arlequin 3.5 was used [46]. The advantage of this method is that it is sensitive to population samples with a common history and substructure. The running parameters are set as follows: 20,000 coalescent simulations and 100 simulated demes. Moreover, the number of simulated groups was based on the results of STRUCTURE. Those with gene frequencies below 0.05 or above 0.95 were excluded from the final results. Loci outside the 95% confidence interval were regarded as outlier loci. In the second approach, a Bayesian method in BayeScan 2.1 was used [49]. The advantage of BayeScan is that it allows for population samples with different demographic histories and different extents of genetic drift [50]. The running parameters are set as follows: a sample size of 5000, a thinning interval of 10, a burn-in of 50,000 iterations, 20 pilot runs with a run length of 5000, and prior odds of 10,000. Here, loci with posterior probability above 0.76 were regarded as outlier loci. Associations between environmental variables and outlier loci were further conducted using LFMM 1.2 [51] to identify potential EAL. A total of 10,000 sweeps and 1000 burn-in sweeps were set as running parameters, and the number of latent factors was obtained from the results of STRUCTURE based on neutral loci (excluding all suspected outlier loci identified by Arlequin and BayeScan). The cut-off values of EAL in LFMM were set as |z| values above 3 according to the study of Vangestel et al. [52] and *P* values below 0.001. Linkage disequilibrium analysis were performed in ARLEQUIN 3.5 [46], and cut-off values were set as D’ and r^2^ values above than 0.8.

Considering that the adaptive potential of a species is mainly determined by its ability to produce adaptive genes and transmit them to other populations [8, 25], we used the genetic differentiation index (*F*_ST_) based on adaptive loci and gene flow (*N*_*m*_) based on neutral loci to indicate these two abilities. Here, we devised a new index, i.e., LAI, to measure the adaptability of species. LAI = *F*_ST(E)_ × *N*_*m*_ *= F*_ST(E)_/4 (1/*F*_ST(N)_ − 1), where *F*_ST(E)_ is the *F*_ST_ value of EAL, *N*_*m*_ is the gene flow among populations, and *F*_ST(N)_ is the *F*_ST_ value of neutral loci. Although the value of *N*_*m*_ is not exactly equal to 1/4 (1/*F*_ST(N)_ − 1) [53], it can roughly reflect the level of gene flow among populations. To investigate the adaptability of species, climatically suitable areas were reconstructed by ecological niche modeling in Maxent 3.4.1 [54]. Information on the geographic distribution of *P. bungeana* was based on a set of 51 presence points covering the entire distribution range: 16 points were obtained from Yang et al. [35], 25 from Zhou [29], and 10 from sampling sites. To further verify this index, we compared it with other species in the same region, namely, *C. coggygria* and *F. suspensa*. The geographic distribution coordinates of these two species were obtained from Wang et al. [55] and Fu et al. [50], and molecular data were obtained from Miao et al. [18] and Yang et al. [3]. The running parameters of ecological niche modeling were set according to the study of Fu et al. [56], but logistic probabilities were used for outputs. Logistic probabilities above 0.5 were taken as climatically suitable areas based on the study of Bai et al. [57].

## Additional files

Additional file 1: The outlier loci identified by BayeScan and Arlequin. (DOC 110 kb)

Additional file 2: Gene frequencies per allele of 430 alleles for each population. (DOCX 57 kb)

Additional file 3: Environmental variables for each location from the WorldClim database. (DOCX 16 kb)

## Data Availability

The datasets supporting the conclusions of this article are included within the article and its additional files. Material samples are available from the corresponding author.

## Abbreviations

AMOVA: Analysis of molecular variance
EAL: Environment-associated loci;
*H*_E_: Nei’s measure of gene diversity
LAI: Landscape adaptation index
*N*_A_: Allele number
PCRs: Polymerase chain reactions
*PPA*: Percentage of polymorphic alleles
RDA: Redundancy analysis
SCoTs: Start codon targeted polymorphisms
