# Supplementary material for: Landscape genetics reveals that adaptive genetic divergence in Pinus bungeana (Pinaceae) is driven by environmental variables relating to ecological habitats
Source: BMC Evol Biol. 2019 Aug 1;19:160. doi: 10.1186/s12862-019-1489-x (PMC6676527; doi:10.1186/s12862-019-1489-x)
Supplement: Supplementary file 1 — The outlier loci identified by BayeScan and Arlequin. (DOC 110 kb) [file 12862_2019_1489_MOESM1_ESM.doc]

**Additional file 1** The outlier loci identified by BayeScan and Arlequin

|  | BayeScan | | | |  | Arlequin | |
| --- | --- | --- | --- | --- | --- | --- | --- |
| Locus | Posterior probability | log10(PO) | q-value | alpha |  | Observe F_ST_ | *F*_ST_ P-value |
| 1-96 | 1.000 | 1000 | 0.000 | 1.823 |  | 0.792 | 0.011 |
| 1-135 |  |  |  |  |  | -0.028 | 0.005 |
| 1-149 | 1.000 | 1000 | 0.000 | 1.817 |  | 0.810 | 0.008 |
| 1-160 | 0.998 | 2.657 | 0.000 | 1.759 |  | 0.688 | 0.044 |
| 1-182 | 1.000 | 1000 | 0.000 | 2.049 |  | 0.931 | 0.001 |
| 1-211 |  |  |  |  |  | 0.659 | 0.041 |
| 1-223 | 0.986 | 1.835 | 0.001 | 1.666 |  |  |  |
| 1-234 | 1.000 | 1000 | 0.000 | 1.993 |  | 0.908 | 0.002 |
| 1-586 |  |  |  |  |  | 0.084 | 0.036 |
| 1-1061 | 0.854 | 0.766 | 0.017 | 1.378 |  |  |  |
| 1-1071 | 0.863 | 0.798 | 0.012 | 1.394 |  |  |  |
| 1-1097 | 0.777 | 0.542 | 0.029 | 1.258 |  |  |  |
| 1-1111 | 0.816 | 0.648 | 0.022 | 1.316 |  |  |  |
| 3-135 |  |  |  |  |  | 0.096 | 0.047 |
| 3-306 |  |  |  |  |  | 0.028 | 0.044 |
| 3-1044 |  |  |  |  |  | 0.056 | 0.041 |
| 6-133 |  |  |  |  |  | 0.027 | 0.005 |
| 6-147 |  |  |  |  |  | 0.045 | 0.021 |
| 6-198 | 0.946 | 1.242 | 0.003 | 1.457 |  |  |  |
| 6-566 |  |  |  |  |  | 0.711 | 0.021 |
| 9-86 | 1.000 | 1000 | 0.000 | 2.140 |  |  |  |
| 9-230 | 1.000 | 1000 | 0.000 | 2.487 |  | 0.827 | 0.023 |
| 9-300 | 0.998 | 2.744 | 0.000 | 1.680 |  |  |  |
| 13-104 | 1.000 | 1000 | 0.000 | 2.175 |  | 0.718 | 0.037 |
| 13-250 |  |  |  |  |  | -0.001 | 0.045 |
| 13-601 | 1.000 | 1000 | 0.000 | 2.491 |  | 0.848 | 0.001 |
| 13-824 |  |  |  |  |  | 0.027 | 0.007 |
| 13-1132 |  |  |  |  |  | 0.080 | 0.033 |
| 19-223 |  |  |  |  |  | 0.003 | 0.008 |
| 19-302 | 1.000 | 1000 | 0.000 | 1.895 |  | 0.824 | 0.006 |
| 19-371 | 1.000 | 1000 | 0.000 | 1.864 |  | 0.792 | 0.010 |
| 19-435 | 1.000 | 1000 | 0.000 | 2.004 |  |  |  |
| 24-85 |  |  |  |  |  | -0.014 | 0.002 |
| 25-199 | 1.000 | 1000 | 0.000 | 1.807 |  |  |  |
| 25-223 |  |  |  |  |  | 0.068 | 0.026 |
| 25-265 | 1.000 | 1000 | 0.000 | 2.161 |  | 0.773 | 0.040 |
| 25-308 | 1.000 | 1000 | 0.000 | 1.878 |  |  |  |
| 25-312 |  |  |  |  |  | 0.639 | 0.044 |
| 25-624 | 0.894 | 0.924 | 0.007 | 1.534 |  | 0.737 | 0.018 |
| 25-709 | 1.000 | 1000 | 0.000 | 2.286 |  |  |  |
| 28-65 |  |  |  |  |  | 0.025 | 0.004 |
| 28-108 | 0.998 | 2.657 | 0.000 | 2.003 |  | 0.915 | 0.002 |
| 28-129 |  |  |  |  |  | 0.043 | 0.008 |
| 28-208 | 1.000 | 1000 | 0.000 | 2.087 |  | 0.785 | 0.036 |
| 28-276 | 1.000 | 1000 | 0.000 | 2.090 |  | 0.821 | 0.000 |
| 28-375 | 1.000 | 1000 | 0.000 | 1.733 |  | 0.719 | 0.040 |
| 28-443 | 1.000 | 1000 | 0.000 | 1.724 |  |  |  |
| 28-626 |  |  |  |  |  | 0.030 | 0.014 |
| 31-172 |  |  |  |  |  | 0.082 | 0.039 |
| 31-192 |  |  |  |  |  | 0.050 | 0.039 |
| 31-265 |  |  |  |  |  | -0.005 | 0.002 |
| 31-278 | 1.000 | 1000 | 0.000 | 1.774 |  |  |  |
| 31-389 |  |  |  |  |  | 0.087 | 0.039 |
| 31-398 |  |  |  |  |  | 0.060 | 0.048 |
| 31-654 |  |  |  |  |  | 0.690 | 0.028 |
| 32-95 |  |  |  |  |  | 0.004 | 0.001 |
| 32-145 |  |  |  |  |  | 0.094 | 0.044 |
| 32-211 |  |  |  |  |  | 0.041 | 0.007 |
| 32-238 |  |  |  |  |  | 0.061 | 0.014 |
| 32-327 |  |  |  |  |  | 0.020 | 0.002 |
| 36-85 |  |  |  |  |  | 0.009 | 0.001 |
| 36-161 |  |  |  |  |  | 0.044 | 0.024 |
| 36-381 |  |  |  |  |  | 0.092 | 0.044 |
