# Supplementary material for: Landscape genetics reveals that adaptive genetic divergence in Pinus bungeana (Pinaceae) is driven by environmental variables relating to ecological habitats
Source: BMC Evol Biol. 2019 Aug 1;19:160. doi: 10.1186/s12862-019-1489-x (PMC6676527; doi:10.1186/s12862-019-1489-x)
Supplement: Supplementary file 2 — Gene frequencies per allele of 430 alleles for each population. (DOCX 57 kb) [file 12862_2019_1489_MOESM2_ESM.docx]

**Additional file 2** Gene frequencies per allele of 430 alleles for each population

| Locus | Allele frequency | | | | | | | | | |
| --- | --- | --- | --- | --- | --- | --- | --- | --- | --- | --- |
|  | 1.SXNN | 2.GXLG | 3.SXLJ | 4.SXWZ | 5.SXWJ | 6.HNSN | 7.SXWL | 8.GSMJ | 9.SCGP | 10.HBLJ |
| 1-96 | 0.824 | 1.000 | 0.882 | 1.000 | 1.000 | 1.000 | 0.875 | 0.947 | 0.000 | 0.111 |
| 1-119 | 0.471 | 0.875 | 0.882 | 0.938 | 0.556 | 0.579 | 0.250 | 0.579 | 0.000 | 0.056 |
| 1-135 | 0.941 | 1.000 | 1.000 | 1.000 | 1.000 | 0.947 | 1.000 | 0.947 | 0.944 | 0.944 |
| 1-149 | 0.941 | 1.000 | 1.000 | 0.938 | 1.000 | 1.000 | 0.813 | 0.895 | 0.000 | 0.111 |
| 1-160 | 0.941 | 1.000 | 0.824 | 0.688 | 1.000 | 1.000 | 0.938 | 0.684 | 0.000 | 0.111 |
| 1-182 | 0.941 | 1.000 | 1.000 | 1.000 | 1.000 | 1.000 | 1.000 | 1.000 | 0.000 | 0.111 |
| 1-195 | 0.059 | 0.063 | 0.000 | 0.000 | 0.000 | 0.000 | 0.000 | 0.000 | 0.000 | 0.000 |
| 1-204 | 0.000 | 0.000 | 0.176 | 0.250 | 0.667 | 0.632 | 0.063 | 0.158 | 0.000 | 0.000 |
| 1-211 | 0.000 | 0.250 | 0.118 | 0.063 | 0.889 | 0.895 | 0.000 | 0.158 | 0.000 | 0.000 |
| 1-217 | 0.000 | 0.125 | 0.118 | 0.000 | 0.000 | 0.000 | 0.000 | 0.000 | 0.000 | 0.000 |
| 1-223 | 0.941 | 0.813 | 0.765 | 0.438 | 1.000 | 0.947 | 0.625 | 0.684 | 0.000 | 0.000 |
| 1-234 | 0.941 | 1.000 | 1.000 | 1.000 | 1.000 | 0.947 | 1.000 | 1.000 | 0.000 | 0.111 |
| 1-249 | 0.000 | 0.000 | 0.059 | 0.000 | 0.111 | 0.000 | 0.000 | 0.000 | 0.000 | 0.000 |
| 1-256 | 0.000 | 0.000 | 0.000 | 0.000 | 0.111 | 0.000 | 0.000 | 0.000 | 0.000 | 0.000 |
| 1-264 | 0.118 | 0.500 | 0.235 | 0.125 | 0.611 | 0.684 | 0.000 | 0.053 | 0.000 | 0.000 |
| 1-277 | 0.000 | 0.000 | 0.000 | 0.063 | 0.000 | 0.000 | 0.063 | 0.000 | 0.000 | 0.000 |
| 1-283 | 0.059 | 0.000 | 0.059 | 0.063 | 0.444 | 0.421 | 0.000 | 0.000 | 0.000 | 0.000 |
| 1-288 | 0.000 | 0.063 | 0.176 | 0.000 | 0.000 | 0.000 | 0.000 | 0.000 | 0.000 | 0.000 |
| 1-306 | 0.000 | 0.000 | 0.529 | 0.250 | 0.889 | 0.947 | 0.375 | 0.316 | 0.000 | 0.278 |
| 1-312 | 0.235 | 0.625 | 0.647 | 0.500 | 0.944 | 0.684 | 0.188 | 0.211 | 0.000 | 0.000 |
| 1-338 | 0.059 | 0.000 | 0.059 | 0.000 | 0.167 | 0.000 | 0.000 | 0.000 | 0.000 | 0.000 |
| 1-378 | 0.000 | 0.000 | 0.059 | 0.000 | 0.000 | 0.000 | 0.000 | 0.000 | 0.000 | 0.000 |
| 1-457 | 0.000 | 0.000 | 0.000 | 0.063 | 0.000 | 0.000 | 0.000 | 0.000 | 0.000 | 0.000 |
| 1-586 | 0.000 | 0.250 | 0.353 | 0.188 | 0.111 | 0.158 | 0.188 | 0.053 | 0.000 | 0.000 |
| 1-1061 | 0.647 | 1.000 | 1.000 | 1.000 | 0.389 | 0.526 | 0.000 | 0.316 | 0.778 | 0.667 |
| 1-1071 | 0.647 | 1.000 | 1.000 | 1.000 | 0.389 | 0.526 | 0.000 | 0.316 | 0.778 | 0.667 |
| 1-1097 | 0.647 | 1.000 | 1.000 | 1.000 | 0.389 | 0.526 | 0.000 | 0.316 | 0.778 | 0.667 |
| 1-1111 | 0.647 | 1.000 | 1.000 | 1.000 | 0.389 | 0.526 | 0.000 | 0.316 | 0.778 | 0.667 |
| 3-76 | 0.000 | 0.000 | 0.059 | 0.000 | 0.000 | 0.000 | 0.000 | 0.000 | 0.000 | 0.000 |
| 3-86 | 0.000 | 0.000 | 0.059 | 0.000 | 0.222 | 0.263 | 0.063 | 0.368 | 0.111 | 0.056 |
| 3-135 | 0.059 | 0.250 | 0.118 | 0.375 | 0.056 | 0.000 | 0.188 | 0.368 | 0.000 | 0.111 |
| 3-146 | 0.059 | 0.000 | 0.118 | 0.000 | 0.000 | 0.000 | 0.000 | 0.526 | 0.000 | 0.000 |
| 3-158 | 0.118 | 0.063 | 0.118 | 0.063 | 0.000 | 0.000 | 0.188 | 0.474 | 0.000 | 0.000 |
| 3-164 | 0.000 | 0.250 | 0.235 | 0.000 | 0.000 | 0.000 | 0.188 | 0.000 | 0.000 | 0.000 |
| 3-168 | 0.059 | 0.000 | 0.000 | 0.063 | 0.000 | 0.000 | 0.000 | 0.474 | 0.000 | 0.000 |
| 3-177 | 0.471 | 0.375 | 0.529 | 0.063 | 0.611 | 0.368 | 0.313 | 0.947 | 0.111 | 0.167 |
| 3-186 | 0.059 | 0.125 | 0.235 | 0.125 | 0.111 | 0.053 | 0.125 | 0.684 | 0.056 | 0.222 |
| 3-198 | 0.176 | 0.000 | 0.294 | 0.063 | 0.222 | 0.053 | 0.313 | 0.737 | 0.056 | 0.000 |
| 3-210 | 0.824 | 1.000 | 0.941 | 0.875 | 1.000 | 1.000 | 0.625 | 1.000 | 0.944 | 1.000 |
| 3-217 | 0.000 | 0.000 | 0.059 | 0.000 | 0.000 | 0.000 | 0.000 | 0.000 | 0.000 | 0.000 |
| 3-227 | 0.706 | 0.563 | 0.941 | 0.750 | 1.000 | 0.947 | 0.563 | 1.000 | 0.667 | 0.667 |
| 3-234 | 0.059 | 0.063 | 0.118 | 0.250 | 0.056 | 0.000 | 0.000 | 0.053 | 0.000 | 0.000 |
| 3-240 | 0.000 | 0.000 | 0.059 | 0.000 | 0.000 | 0.000 | 0.125 | 0.316 | 0.000 | 0.000 |
| 3-248 | 0.000 | 0.000 | 0.118 | 0.000 | 0.000 | 0.000 | 0.000 | 0.053 | 0.000 | 0.000 |
| 3-256 | 0.000 | 0.000 | 0.059 | 0.063 | 0.056 | 0.000 | 0.188 | 0.316 | 0.056 | 0.000 |
| 3-263 | 0.000 | 1.000 | 0.118 | 0.000 | 0.333 | 0.368 | 0.000 | 0.474 | 0.056 | 0.278 |
| 3-272 | 0.294 | 0.063 | 0.412 | 0.250 | 0.611 | 0.421 | 0.250 | 0.684 | 0.056 | 0.000 |
| 3-281 | 0.118 | 0.000 | 0.176 | 0.125 | 0.222 | 0.053 | 0.000 | 0.000 | 0.333 | 0.500 |
| 3-288 | 0.000 | 0.000 | 0.000 | 0.000 | 0.000 | 0.000 | 0.063 | 0.105 | 0.000 | 0.000 |
| 3-295 | 0.000 | 0.000 | 0.059 | 0.000 | 0.000 | 0.000 | 0.000 | 0.053 | 0.000 | 0.000 |
| 3-306 | 0.882 | 0.875 | 1.000 | 0.938 | 1.000 | 0.947 | 1.000 | 1.000 | 1.000 | 1.000 |
| 3-312 | 0.059 | 0.000 | 0.000 | 0.000 | 0.000 | 0.000 | 0.000 | 0.000 | 0.000 | 0.000 |
| 3-318 | 0.176 | 0.500 | 0.706 | 0.875 | 1.000 | 1.000 | 0.438 | 0.737 | 0.778 | 0.833 |
| 3-322 | 0.000 | 0.000 | 0.000 | 0.000 | 0.000 | 0.000 | 0.000 | 0.000 | 0.000 | 0.000 |
| 3-328 | 0.000 | 0.000 | 0.059 | 0.000 | 0.000 | 0.000 | 0.000 | 0.000 | 0.000 | 0.000 |
| 3-364 | 0.000 | 0.000 | 0.000 | 0.000 | 0.556 | 0.211 | 0.063 | 0.105 | 0.000 | 0.000 |
| 3-376 | 0.059 | 0.000 | 0.000 | 0.000 | 0.000 | 0.000 | 0.000 | 0.000 | 0.000 | 0.000 |
| 3-394 | 0.000 | 0.000 | 0.000 | 0.000 | 0.167 | 0.105 | 0.000 | 0.053 | 0.056 | 0.222 |
| 3-398 | 0.000 | 0.000 | 0.353 | 0.000 | 0.167 | 0.000 | 0.000 | 0.158 | 0.000 | 0.000 |
| 3-406 | 0.000 | 0.000 | 0.059 | 0.000 | 0.000 | 0.000 | 0.000 | 0.000 | 0.000 | 0.000 |
| 3-420 | 0.000 | 0.000 | 0.000 | 0.000 | 0.000 | 0.000 | 0.000 | 0.053 | 0.000 | 0.000 |
| 3-466 | 0.059 | 0.125 | 0.000 | 0.125 | 0.000 | 0.053 | 0.000 | 0.000 | 0.000 | 0.000 |
| 3-530 | 0.059 | 0.063 | 0.118 | 0.188 | 0.000 | 0.000 | 0.000 | 0.000 | 0.000 | 0.000 |
| 3-586 | 0.824 | 0.813 | 0.882 | 1.000 | 1.000 | 0.737 | 0.750 | 0.737 | 0.833 | 0.333 |
| 3-610 | 0.059 | 0.000 | 0.000 | 0.000 | 0.500 | 0.368 | 0.000 | 0.000 | 0.333 | 0.444 |
| 3-1029 | 0.059 | 0.000 | 0.000 | 0.000 | 0.000 | 0.000 | 0.000 | 0.000 | 0.056 | 0.000 |
| 3-1044 | 0.059 | 0.000 | 0.000 | 0.063 | 0.000 | 0.053 | 0.063 | 0.000 | 0.222 | 0.111 |
| 3-1056 | 0.059 | 0.000 | 0.000 | 0.000 | 0.000 | 0.000 | 0.000 | 0.000 | 0.056 | 0.000 |
| 6-61 | 0.059 | 0.188 | 0.000 | 0.000 | 0.000 | 0.000 | 0.063 | 0.105 | 0.611 | 0.222 |
| 6-76 | 0.059 | 0.000 | 0.059 | 0.000 | 0.000 | 0.000 | 0.000 | 0.000 | 0.000 | 0.000 |
| 6-107 | 0.059 | 0.000 | 0.000 | 0.000 | 0.000 | 0.000 | 0.000 | 0.000 | 0.000 | 0.111 |
| 6-133 | 0.706 | 0.813 | 0.529 | 0.813 | 0.667 | 0.579 | 0.500 | 0.737 | 0.833 | 0.833 |
| 6-147 | 0.176 | 0.000 | 0.059 | 0.125 | 0.222 | 0.053 | 0.063 | 0.000 | 0.000 | 0.000 |
| 6-157 | 0.118 | 0.000 | 0.000 | 0.000 | 0.000 | 0.000 | 0.063 | 0.053 | 0.000 | 0.111 |
| 6-182 | 0.529 | 0.625 | 0.471 | 0.563 | 0.667 | 0.526 | 0.125 | 0.316 | 0.111 | 0.111 |
| 6-198 | 0.353 | 0.063 | 0.059 | 0.063 | 1.000 | 0.947 | 0.063 | 0.421 | 0.833 | 0.500 |
| 6-204 | 0.059 | 0.000 | 0.000 | 0.000 | 0.500 | 0.105 | 0.000 | 0.000 | 0.111 | 0.111 |
| 6-217 | 0.647 | 0.688 | 0.412 | 0.750 | 0.944 | 0.947 | 0.313 | 0.789 | 1.000 | 0.889 |
| 6-227 | 0.059 | 0.000 | 0.059 | 0.000 | 0.000 | 0.000 | 0.000 | 0.000 | 0.056 | 0.056 |
| 6-234 | 0.588 | 0.563 | 0.353 | 0.500 | 0.556 | 0.474 | 0.000 | 0.158 | 0.000 | 0.000 |
| 6-246 | 0.059 | 0.000 | 0.000 | 0.000 | 0.056 | 0.053 | 0.000 | 0.000 | 0.056 | 0.000 |
| 6-268 | 0.176 | 0.063 | 0.059 | 0.063 | 0.833 | 0.632 | 0.000 | 0.211 | 0.389 | 0.000 |
| 6-304 | 0.118 | 0.000 | 0.059 | 0.000 | 0.778 | 0.474 | 0.000 | 0.053 | 0.500 | 0.278 |
| 6-317 | 0.059 | 0.000 | 0.059 | 0.000 | 0.000 | 0.000 | 0.000 | 0.053 | 0.000 | 0.000 |
| 6-327 | 1.000 | 1.000 | 1.000 | 1.000 | 1.000 | 1.000 | 1.000 | 1.000 | 1.000 | 0.833 |
| 6-337 | 0.059 | 0.000 | 0.000 | 0.000 | 0.000 | 0.000 | 0.000 | 0.000 | 0.000 | 0.167 |
| 6-358 | 0.059 | 0.000 | 0.000 | 0.000 | 0.056 | 0.000 | 0.000 | 0.000 | 0.000 | 0.000 |
| 6-398 | 0.118 | 0.000 | 0.000 | 0.000 | 0.500 | 0.316 | 0.000 | 0.000 | 0.056 | 0.000 |
| 6-427 | 0.059 | 0.000 | 0.000 | 0.000 | 0.056 | 0.053 | 0.000 | 0.000 | 0.000 | 0.000 |
| 6-538 | 0.059 | 0.000 | 0.059 | 0.000 | 0.000 | 0.158 | 0.000 | 0.000 | 0.056 | 0.056 |
| 6-566 | 0.059 | 0.000 | 0.000 | 0.000 | 0.611 | 0.947 | 0.000 | 0.000 | 0.111 | 0.000 |
| 6-586 | 0.059 | 0.000 | 0.000 | 0.000 | 0.000 | 0.053 | 0.000 | 0.000 | 0.000 | 0.000 |
| 6-596 | 0.059 | 0.000 | 0.000 | 0.000 | 0.167 | 0.158 | 0.000 | 0.000 | 0.000 | 0.000 |
| 6-670 | 0.059 | 0.000 | 0.000 | 0.000 | 0.056 | 0.105 | 0.000 | 0.000 | 0.000 | 0.000 |
| 6-735 | 0.059 | 0.000 | 0.000 | 0.000 | 0.056 | 0.000 | 0.000 | 0.000 | 0.000 | 0.000 |
| 6-974 | 0.059 | 0.000 | 0.000 | 0.000 | 0.000 | 0.000 | 0.000 | 0.000 | 0.000 | 0.056 |
| 6-989 | 0.059 | 0.000 | 0.000 | 0.000 | 0.000 | 0.053 | 0.000 | 0.000 | 0.000 | 0.000 |
| 6-1062 | 0.059 | 0.000 | 0.059 | 0.000 | 0.000 | 0.000 | 0.000 | 0.000 | 0.000 | 0.000 |
| 9-86 | 1.000 | 1.000 | 0.706 | 1.000 | 0.053 | 0.105 | 0.063 | 0.105 | 0.056 | 0.278 |
| 9-134 | 0.235 | 0.250 | 0.000 | 0.063 | 0.000 | 0.000 | 0.000 | 0.000 | 0.000 | 0.000 |
| 9-160 | 0.118 | 0.000 | 0.000 | 0.000 | 0.000 | 0.000 | 0.000 | 0.000 | 0.000 | 0.000 |
| 9-183 | 0.176 | 0.000 | 0.000 | 0.000 | 0.000 | 0.000 | 0.000 | 0.000 | 0.000 | 0.000 |
| 9-230 | 0.882 | 1.000 | 0.588 | 1.000 | 0.000 | 0.211 | 0.000 | 0.000 | 0.000 | 0.000 |
| 9-300 | 0.941 | 1.000 | 0.647 | 1.000 | 0.053 | 0.737 | 0.125 | 0.211 | 0.722 | 0.167 |
| 9-358 | 0.235 | 0.000 | 0.000 | 0.000 | 0.000 | 0.000 | 0.000 | 0.000 | 0.000 | 0.056 |
| 9-375 | 0.118 | 0.000 | 0.000 | 0.000 | 0.000 | 0.000 | 0.000 | 0.000 | 0.000 | 0.000 |
| 9-400 | 0.118 | 0.000 | 0.000 | 0.000 | 0.000 | 0.158 | 0.000 | 0.000 | 0.722 | 0.056 |
| 9-411 | 0.118 | 0.000 | 0.000 | 0.000 | 0.000 | 0.053 | 0.000 | 0.000 | 0.000 | 0.056 |
| 9-420 | 0.059 | 0.125 | 0.000 | 0.000 | 0.000 | 0.000 | 0.000 | 0.000 | 0.222 | 0.000 |
| 9-441 | 0.059 | 0.000 | 0.000 | 0.000 | 0.000 | 0.000 | 0.000 | 0.000 | 0.000 | 0.000 |
| 9-484 | 0.059 | 0.000 | 0.059 | 0.000 | 0.000 | 0.000 | 0.000 | 0.000 | 0.000 | 0.000 |
| 9-493 | 0.059 | 0.000 | 0.000 | 0.000 | 0.053 | 0.000 | 0.000 | 0.000 | 0.000 | 0.000 |
| 9-499 | 0.059 | 0.000 | 0.000 | 0.000 | 0.000 | 0.000 | 0.000 | 0.000 | 0.000 | 0.056 |
| 9-504 | 0.059 | 0.000 | 0.000 | 0.000 | 0.000 | 0.000 | 0.000 | 0.000 | 0.000 | 0.056 |
| 9-511 | 0.059 | 0.000 | 0.000 | 0.000 | 0.000 | 0.000 | 0.000 | 0.000 | 0.000 | 0.056 |
| 9-526 | 0.059 | 0.000 | 0.059 | 0.000 | 0.000 | 0.000 | 0.000 | 0.000 | 0.000 | 0.000 |
| 9-545 | 0.059 | 0.000 | 0.000 | 0.000 | 0.000 | 0.105 | 0.000 | 0.000 | 0.000 | 0.000 |
| 9-552 | 0.059 | 0.000 | 0.000 | 0.000 | 0.000 | 0.053 | 0.000 | 0.000 | 0.000 | 0.000 |
| 9-556 | 0.176 | 0.000 | 0.000 | 0.000 | 0.000 | 0.000 | 0.000 | 0.000 | 0.000 | 0.000 |
| 9-565 | 0.059 | 0.000 | 0.000 | 0.000 | 0.000 | 0.000 | 0.000 | 0.000 | 0.111 | 0.000 |
| 9-603 | 0.059 | 0.000 | 0.000 | 0.000 | 0.000 | 0.000 | 0.000 | 0.000 | 0.000 | 0.000 |
| 9-623 | 0.059 | 0.000 | 0.000 | 0.000 | 0.000 | 0.316 | 0.000 | 0.105 | 0.000 | 0.000 |
| 9-663 | 0.059 | 0.000 | 0.059 | 0.000 | 0.000 | 0.000 | 0.000 | 0.000 | 0.000 | 0.000 |
| 9-670 | 0.059 | 0.000 | 0.000 | 0.000 | 0.000 | 0.053 | 0.000 | 0.000 | 0.000 | 0.000 |
| 9-713 | 0.059 | 0.125 | 0.000 | 0.000 | 0.000 | 0.000 | 0.000 | 0.000 | 0.000 | 0.000 |
| 9-720 | 0.706 | 0.875 | 0.706 | 0.938 | 0.316 | 0.842 | 0.438 | 0.421 | 0.722 | 1.000 |
| 9-766 | 0.059 | 0.000 | 0.000 | 0.000 | 0.000 | 0.000 | 0.063 | 0.000 | 0.000 | 0.000 |
| 9-788 | 0.059 | 0.000 | 0.000 | 0.000 | 0.000 | 0.053 | 0.000 | 0.000 | 0.056 | 0.000 |
| 9-813 | 0.118 | 0.500 | 0.765 | 0.688 | 0.474 | 0.947 | 0.500 | 0.737 | 0.833 | 0.778 |
| 9-823 | 0.118 | 0.188 | 0.353 | 0.375 | 0.053 | 0.526 | 0.438 | 0.579 | 0.778 | 0.333 |
| 9-869 | 0.059 | 0.000 | 0.000 | 0.000 | 0.000 | 0.000 | 0.000 | 0.000 | 0.056 | 0.000 |
| 9-925 | 0.059 | 0.063 | 0.000 | 0.000 | 0.000 | 0.000 | 0.000 | 0.000 | 0.000 | 0.000 |
| 9-950 | 0.059 | 0.000 | 0.000 | 0.000 | 0.000 | 0.053 | 0.000 | 0.000 | 0.000 | 0.000 |
| 9-966 | 0.059 | 0.188 | 0.353 | 0.000 | 0.842 | 0.789 | 0.688 | 0.737 | 0.000 | 0.667 |
| 9-1004 | 0.059 | 0.125 | 0.059 | 0.000 | 0.000 | 0.000 | 0.000 | 0.000 | 0.111 | 0.056 |
| 9-1023 | 0.059 | 0.000 | 0.059 | 0.125 | 0.000 | 0.000 | 0.000 | 0.000 | 0.000 | 0.000 |
| 9-1061 | 0.118 | 0.000 | 0.000 | 0.000 | 0.000 | 0.053 | 0.000 | 0.000 | 0.000 | 0.000 |
| 9-1104 | 0.059 | 0.000 | 0.000 | 0.000 | 0.000 | 0.105 | 0.063 | 0.053 | 0.056 | 0.000 |
| 9-1114 | 0.059 | 0.000 | 0.000 | 0.000 | 0.053 | 0.000 | 0.188 | 0.000 | 0.056 | 0.056 |
| 9-1150 | 0.059 | 0.000 | 0.000 | 0.000 | 0.000 | 0.000 | 0.000 | 0.000 | 0.056 | 0.000 |
| 9-1173 | 0.059 | 0.000 | 0.000 | 0.000 | 0.000 | 0.000 | 0.000 | 0.000 | 0.000 | 0.000 |
| 9-1176 | 0.059 | 0.000 | 0.000 | 0.000 | 0.000 | 0.000 | 0.125 | 0.000 | 0.000 | 0.000 |
| 9-1183 | 0.059 | 0.000 | 0.000 | 0.000 | 0.000 | 0.000 | 0.063 | 0.000 | 0.000 | 0.000 |
| 9-1190 | 0.059 | 0.000 | 0.000 | 0.000 | 0.000 | 0.000 | 0.063 | 0.000 | 0.000 | 0.000 |
| 9-1197 | 0.059 | 0.000 | 0.000 | 0.000 | 0.000 | 0.000 | 0.000 | 0.000 | 0.000 | 0.056 |
| 13-86 | 0.059 | 0.063 | 0.235 | 0.063 | 0.000 | 0.000 | 0.000 | 0.000 | 0.056 | 0.000 |
| 13-92 | 0.824 | 0.750 | 1.000 | 1.000 | 1.000 | 1.000 | 1.000 | 0.895 | 1.000 | 0.444 |
| 13-104 | 0.353 | 0.000 | 0.882 | 0.000 | 1.000 | 1.000 | 1.000 | 0.947 | 0.889 | 0.389 |
| 13-145 | 0.647 | 0.188 | 1.000 | 1.000 | 1.000 | 1.000 | 1.000 | 0.947 | 0.889 | 0.389 |
| 13-183 | 0.529 | 0.063 | 0.941 | 0.938 | 0.526 | 0.474 | 0.125 | 0.211 | 0.389 | 0.056 |
| 13-235 | 0.176 | 0.000 | 0.000 | 0.000 | 0.000 | 0.000 | 0.000 | 0.000 | 0.000 | 0.000 |
| 13-250 | 0.882 | 1.000 | 1.000 | 1.000 | 1.000 | 1.000 | 1.000 | 0.947 | 0.944 | 0.944 |
| 13-300 | 0.118 | 0.000 | 0.000 | 0.063 | 0.000 | 0.000 | 0.000 | 0.000 | 0.000 | 0.000 |
| 13-334 | 0.882 | 0.813 | 0.882 | 0.750 | 0.895 | 1.000 | 0.813 | 0.579 | 0.833 | 0.389 |
| 13-395 | 0.118 | 0.000 | 0.000 | 0.000 | 0.000 | 0.000 | 0.000 | 0.000 | 0.000 | 0.000 |
| 13-418 | 0.706 | 0.875 | 1.000 | 0.875 | 1.000 | 1.000 | 1.000 | 0.947 | 0.944 | 0.944 |
| 13-449 | 0.941 | 1.000 | 0.765 | 0.375 | 0.316 | 0.368 | 0.125 | 0.105 | 0.278 | 0.056 |
| 13-489 | 0.118 | 0.000 | 0.824 | 0.250 | 0.895 | 0.737 | 0.813 | 0.474 | 0.889 | 0.611 |
| 13-524 | 0.059 | 0.000 | 0.000 | 0.000 | 0.000 | 0.000 | 0.000 | 0.000 | 0.000 | 0.000 |
| 13-601 | 0.118 | 0.000 | 1.000 | 0.938 | 1.000 | 1.000 | 1.000 | 0.947 | 0.944 | 1.000 |
| 13-632 | 0.529 | 1.000 | 0.706 | 0.500 | 0.211 | 0.263 | 0.250 | 0.053 | 0.333 | 0.167 |
| 13-636 | 0.235 | 0.000 | 0.000 | 0.000 | 0.000 | 0.000 | 0.000 | 0.000 | 0.000 | 0.000 |
| 13-720 | 0.059 | 0.000 | 0.176 | 0.125 | 0.000 | 0.158 | 0.000 | 0.053 | 0.111 | 0.389 |
| 13-812 | 0.059 | 0.000 | 0.000 | 0.000 | 0.000 | 0.474 | 0.125 | 0.053 | 0.222 | 0.278 |
| 13-824 | 0.059 | 0.000 | 0.059 | 0.000 | 0.000 | 0.105 | 0.125 | 0.053 | 0.222 | 0.111 |
| 13-845 | 0.059 | 0.000 | 0.000 | 0.000 | 0.000 | 0.000 | 0.063 | 0.000 | 0.000 | 0.000 |
| 13-869 | 0.059 | 0.000 | 0.059 | 0.000 | 0.000 | 0.000 | 0.000 | 0.000 | 0.056 | 0.000 |
| 13-913 | 0.059 | 0.000 | 0.000 | 0.000 | 0.000 | 0.000 | 0.063 | 0.000 | 0.000 | 0.000 |
| 13-950 | 0.059 | 0.000 | 0.000 | 0.000 | 0.000 | 0.053 | 0.000 | 0.000 | 0.000 | 0.000 |
| 13-961 | 0.059 | 0.000 | 0.000 | 0.000 | 0.000 | 0.000 | 0.000 | 0.000 | 0.000 | 0.000 |
| 13-966 | 0.059 | 0.000 | 0.000 | 0.000 | 0.158 | 0.474 | 0.313 | 0.105 | 0.000 | 0.222 |
| 13-1043 | 0.059 | 0.000 | 0.000 | 0.000 | 0.000 | 0.053 | 0.000 | 0.000 | 0.000 | 0.000 |
| 13-1056 | 0.059 | 0.000 | 0.000 | 0.000 | 0.053 | 0.000 | 0.000 | 0.000 | 0.000 | 0.000 |
| 13-1083 | 0.059 | 0.000 | 0.000 | 0.000 | 0.000 | 0.053 | 0.000 | 0.000 | 0.000 | 0.000 |
| 13-1103 | 0.059 | 0.000 | 0.000 | 0.000 | 0.000 | 0.000 | 0.000 | 0.000 | 0.000 | 0.056 |
| 13-1132 | 0.059 | 0.000 | 0.176 | 0.063 | 0.053 | 0.263 | 0.438 | 0.105 | 0.111 | 0.278 |
| 19-82 | 0.647 | 0.000 | 0.000 | 0.000 | 0.053 | 0.000 | 0.000 | 0.000 | 0.111 | 0.111 |
| 19-88 | 0.059 | 0.000 | 0.000 | 0.000 | 0.053 | 0.000 | 0.000 | 0.000 | 0.000 | 0.000 |
| 19-96 | 0.059 | 0.000 | 0.000 | 0.000 | 0.053 | 0.000 | 0.000 | 0.000 | 0.000 | 0.000 |
| 19-102 | 0.059 | 0.000 | 0.000 | 0.000 | 0.000 | 0.000 | 0.000 | 0.000 | 0.000 | 0.000 |
| 19-109 | 0.176 | 0.000 | 0.000 | 0.000 | 0.000 | 0.000 | 0.000 | 0.000 | 0.000 | 0.000 |
| 19-123 | 0.059 | 0.000 | 0.000 | 0.000 | 0.053 | 0.000 | 0.000 | 0.000 | 0.000 | 0.000 |
| 19-132 | 0.588 | 0.000 | 0.000 | 0.000 | 0.421 | 0.053 | 0.000 | 0.000 | 0.000 | 0.000 |
| 19-171 | 0.059 | 0.000 | 0.000 | 0.000 | 0.000 | 0.000 | 0.000 | 0.000 | 0.000 | 0.000 |
| 19-179 | 0.118 | 0.000 | 0.000 | 0.063 | 0.474 | 0.158 | 0.000 | 0.000 | 0.000 | 0.000 |
| 19-191 | 0.059 | 0.125 | 0.059 | 0.063 | 0.000 | 0.000 | 0.000 | 0.000 | 0.000 | 0.000 |
| 19-196 | 0.118 | 0.000 | 0.000 | 0.063 | 0.526 | 0.211 | 0.063 | 0.158 | 0.000 | 0.000 |
| 19-223 | 1.000 | 0.875 | 1.000 | 1.000 | 0.947 | 1.000 | 0.938 | 0.947 | 1.000 | 1.000 |
| 19-234 | 0.059 | 0.063 | 0.000 | 0.000 | 0.000 | 0.000 | 0.000 | 0.000 | 0.000 | 0.000 |
| 19-258 | 0.059 | 0.000 | 0.000 | 0.000 | 0.000 | 0.053 | 0.000 | 0.000 | 0.000 | 0.000 |
| 19-271 | 0.059 | 0.000 | 0.000 | 0.000 | 0.053 | 0.000 | 0.000 | 0.000 | 0.000 | 0.000 |
| 19-278 | 0.059 | 0.000 | 0.000 | 0.000 | 0.000 | 0.000 | 0.000 | 0.000 | 0.000 | 0.000 |
| 19-287 | 0.059 | 0.000 | 0.294 | 0.000 | 0.158 | 0.158 | 0.563 | 0.053 | 0.000 | 0.000 |
| 19-302 | 0.941 | 0.938 | 1.000 | 1.000 | 0.947 | 0.789 | 0.938 | 0.947 | 0.000 | 0.000 |
| 19-315 | 0.059 | 0.000 | 0.000 | 0.000 | 0.000 | 0.000 | 0.000 | 0.000 | 0.000 | 0.000 |
| 19-344 | 0.059 | 0.000 | 0.000 | 0.000 | 0.053 | 0.000 | 0.000 | 0.053 | 0.000 | 0.000 |
| 19-349 | 0.118 | 0.063 | 0.059 | 0.125 | 0.000 | 0.000 | 0.000 | 0.000 | 0.000 | 0.000 |
| 19-356 | 0.059 | 0.000 | 0.000 | 0.000 | 0.000 | 0.000 | 0.000 | 0.000 | 0.000 | 0.000 |
| 19-362 | 0.059 | 0.000 | 0.059 | 0.000 | 0.000 | 0.000 | 0.000 | 0.000 | 0.000 | 0.000 |
| 19-371 | 0.824 | 0.938 | 1.000 | 1.000 | 0.947 | 0.789 | 0.938 | 0.947 | 0.000 | 0.000 |
| 19-396 | 0.059 | 0.000 | 0.000 | 0.000 | 0.000 | 0.000 | 0.000 | 0.000 | 0.000 | 0.000 |
| 19-409 | 0.059 | 0.000 | 0.000 | 0.000 | 0.000 | 0.000 | 0.000 | 0.000 | 0.000 | 0.000 |
| 19-435 | 0.706 | 0.875 | 0.706 | 0.938 | 0.000 | 0.211 | 0.000 | 0.000 | 0.889 | 1.000 |
| 19-475 | 0.059 | 0.000 | 0.000 | 0.000 | 0.000 | 0.000 | 0.000 | 0.000 | 0.000 | 0.000 |
| 19-484 | 0.118 | 0.000 | 0.000 | 0.000 | 0.000 | 0.000 | 0.000 | 0.000 | 0.000 | 0.000 |
| 19-492 | 0.059 | 0.000 | 0.000 | 0.000 | 0.000 | 0.000 | 0.000 | 0.000 | 0.000 | 0.000 |
| 19-552 | 0.118 | 0.188 | 0.059 | 0.000 | 0.000 | 0.000 | 0.000 | 0.000 | 0.056 | 0.000 |
| 19-749 | 0.118 | 0.063 | 0.000 | 0.000 | 0.000 | 0.000 | 0.000 | 0.000 | 0.000 | 0.000 |
| 24-66 | 0.059 | 0.000 | 0.059 | 0.000 | 0.000 | 0.000 | 0.063 | 0.000 | 0.000 | 0.000 |
| 24-72 | 0.059 | 0.000 | 0.059 | 0.000 | 0.053 | 0.000 | 0.063 | 0.053 | 0.000 | 0.000 |
| 24-85 | 1.000 | 0.938 | 0.941 | 0.938 | 0.947 | 0.947 | 0.875 | 0.895 | 1.000 | 1.000 |
| 24-95 | 0.059 | 0.000 | 0.059 | 0.000 | 0.000 | 0.000 | 0.063 | 0.053 | 0.000 | 0.111 |
| 24-109 | 0.059 | 0.000 | 0.059 | 0.000 | 0.000 | 0.000 | 0.125 | 0.105 | 0.000 | 0.000 |
| 24-116 | 0.059 | 0.000 | 0.059 | 0.000 | 0.053 | 0.000 | 0.000 | 0.053 | 0.000 | 0.000 |
| 24-126 | 0.059 | 0.000 | 0.000 | 0.000 | 0.053 | 0.000 | 0.000 | 0.000 | 0.000 | 0.000 |
| 24-152 | 0.059 | 0.000 | 0.059 | 0.000 | 0.000 | 0.000 | 0.063 | 0.053 | 0.000 | 0.000 |
| 24-215 | 0.059 | 0.000 | 0.000 | 0.000 | 0.000 | 0.000 | 0.063 | 0.053 | 0.000 | 0.000 |
| 24-223 | 0.059 | 0.000 | 0.000 | 0.000 | 0.053 | 0.000 | 0.000 | 0.000 | 0.000 | 0.000 |
| 24-230 | 0.059 | 0.000 | 0.059 | 0.000 | 0.000 | 0.000 | 0.000 | 0.000 | 0.000 | 0.000 |
| 24-421 | 0.059 | 0.000 | 0.000 | 0.000 | 0.000 | 0.000 | 0.000 | 0.053 | 0.000 | 0.000 |
| 24-480 | 0.118 | 0.000 | 0.000 | 0.000 | 0.158 | 0.053 | 0.000 | 0.000 | 0.000 | 0.000 |
| 24-489 | 0.059 | 0.000 | 0.000 | 0.000 | 0.000 | 0.000 | 0.063 | 0.000 | 0.000 | 0.000 |
| 24-534 | 0.059 | 0.063 | 0.000 | 0.000 | 0.000 | 0.000 | 0.000 | 0.000 | 0.000 | 0.000 |
| 24-756 | 0.059 | 0.000 | 0.000 | 0.000 | 0.053 | 0.000 | 0.000 | 0.000 | 0.000 | 0.000 |
| 24-923 | 0.059 | 0.000 | 0.059 | 0.000 | 0.000 | 0.000 | 0.000 | 0.000 | 0.000 | 0.000 |
| 25-63 | 0.059 | 0.000 | 0.000 | 0.000 | 0.053 | 0.000 | 0.000 | 0.000 | 0.000 | 0.000 |
| 25-82 | 0.118 | 0.000 | 0.000 | 0.000 | 0.000 | 0.000 | 0.000 | 0.000 | 0.000 | 0.000 |
| 25-91 | 0.118 | 0.000 | 0.235 | 0.125 | 0.368 | 0.000 | 0.250 | 0.737 | 0.056 | 0.111 |
| 25-96 | 0.059 | 0.000 | 0.000 | 0.063 | 0.053 | 0.000 | 0.000 | 0.000 | 0.000 | 0.000 |
| 25-115 | 0.059 | 0.000 | 0.000 | 0.000 | 0.211 | 0.000 | 0.000 | 0.000 | 0.000 | 0.000 |
| 25-123 | 0.059 | 0.000 | 0.000 | 0.000 | 0.053 | 0.000 | 0.000 | 0.000 | 0.000 | 0.000 |
| 25-138 | 0.059 | 0.000 | 0.000 | 0.000 | 0.053 | 0.000 | 0.000 | 0.000 | 0.000 | 0.000 |
| 25-148 | 0.059 | 0.000 | 0.000 | 0.000 | 0.158 | 0.000 | 0.000 | 0.000 | 0.000 | 0.000 |
| 25-163 | 0.118 | 0.188 | 0.706 | 0.625 | 0.895 | 0.947 | 0.750 | 0.895 | 0.222 | 0.000 |
| 25-199 | 0.882 | 0.813 | 0.588 | 1.000 | 0.895 | 0.947 | 0.125 | 0.474 | 0.000 | 0.000 |
| 25-208 | 0.059 | 0.000 | 0.059 | 0.000 | 0.053 | 0.000 | 0.000 | 0.000 | 0.000 | 0.000 |
| 25-223 | 1.000 | 0.875 | 0.941 | 0.938 | 0.947 | 1.000 | 0.625 | 0.842 | 0.944 | 0.889 |
| 25-238 | 0.059 | 0.000 | 0.000 | 0.063 | 0.421 | 0.000 | 0.000 | 0.000 | 0.000 | 0.000 |
| 25-253 | 0.059 | 0.000 | 0.059 | 0.000 | 0.316 | 0.000 | 0.000 | 0.000 | 0.000 | 0.000 |
| 25-265 | 1.000 | 0.875 | 0.588 | 1.000 | 0.895 | 0.947 | 0.063 | 0.105 | 0.000 | 0.000 |
| 25-276 | 0.059 | 0.000 | 0.000 | 0.000 | 0.053 | 0.000 | 0.000 | 0.000 | 0.000 | 0.000 |
| 25-290 | 0.059 | 0.000 | 0.000 | 0.000 | 0.000 | 0.000 | 0.000 | 0.000 | 0.000 | 0.000 |
| 25-302 | 0.176 | 0.000 | 0.000 | 0.125 | 0.105 | 0.000 | 0.063 | 0.316 | 0.000 | 0.000 |
| 25-308 | 0.529 | 0.688 | 0.765 | 1.000 | 0.421 | 0.000 | 0.000 | 0.053 | 0.000 | 0.000 |
| 25-312 | 0.235 | 0.063 | 0.000 | 0.000 | 0.474 | 0.947 | 0.000 | 0.000 | 0.000 | 0.000 |
| 25-328 | 0.059 | 0.000 | 0.000 | 0.000 | 0.000 | 0.000 | 0.000 | 0.000 | 0.000 | 0.000 |
| 25-349 | 0.059 | 0.000 | 0.118 | 0.125 | 0.053 | 0.000 | 0.000 | 0.000 | 0.000 | 0.000 |
| 25-371 | 0.059 | 0.000 | 0.000 | 0.000 | 0.000 | 0.000 | 0.063 | 0.263 | 0.000 | 0.000 |
| 25-376 | 0.059 | 0.063 | 0.000 | 0.750 | 0.316 | 0.000 | 0.000 | 0.000 | 0.000 | 0.000 |
| 25-391 | 0.059 | 0.063 | 0.412 | 0.125 | 0.158 | 0.000 | 0.000 | 0.000 | 0.000 | 0.000 |
| 25-399 | 0.059 | 0.063 | 0.000 | 0.000 | 0.000 | 0.000 | 0.000 | 0.000 | 0.000 | 0.000 |
| 25-417 | 0.059 | 0.000 | 0.000 | 0.000 | 0.000 | 0.000 | 0.000 | 0.000 | 0.000 | 0.000 |
| 25-426 | 0.059 | 0.000 | 0.000 | 0.000 | 0.000 | 0.000 | 0.000 | 0.000 | 0.000 | 0.000 |
| 25-435 | 0.176 | 0.063 | 0.000 | 0.000 | 0.000 | 0.000 | 0.000 | 0.000 | 0.000 | 0.000 |
| 25-443 | 0.059 | 0.000 | 0.000 | 0.000 | 0.053 | 0.000 | 0.000 | 0.000 | 0.000 | 0.000 |
| 25-508 | 0.059 | 0.563 | 0.000 | 0.000 | 0.000 | 0.000 | 0.000 | 0.000 | 0.000 | 0.000 |
| 25-544 | 0.059 | 0.125 | 0.000 | 0.125 | 0.368 | 0.316 | 0.000 | 0.000 | 0.000 | 0.000 |
| 25-548 | 0.059 | 0.188 | 0.000 | 0.000 | 0.000 | 0.000 | 0.000 | 0.000 | 0.000 | 0.000 |
| 25-591 | 0.059 | 0.063 | 0.000 | 0.000 | 0.000 | 0.000 | 0.000 | 0.000 | 0.000 | 0.000 |
| 25-609 | 0.059 | 0.000 | 0.000 | 0.000 | 0.053 | 0.000 | 0.000 | 0.000 | 0.000 | 0.000 |
| 25-624 | 0.059 | 0.188 | 0.765 | 1.000 | 0.105 | 0.000 | 0.000 | 0.000 | 0.000 | 0.000 |
| 25-661 | 0.059 | 0.000 | 0.000 | 0.000 | 0.000 | 0.000 | 0.000 | 0.000 | 0.000 | 0.000 |
| 25-709 | 0.882 | 0.875 | 0.529 | 1.000 | 0.316 | 0.000 | 0.000 | 0.000 | 0.000 | 0.000 |
| 25-753 | 0.059 | 0.000 | 0.000 | 0.000 | 0.000 | 0.000 | 0.000 | 0.000 | 0.000 | 0.000 |
| 25-785 | 0.059 | 0.000 | 0.000 | 0.000 | 0.000 | 0.000 | 0.000 | 0.000 | 0.000 | 0.000 |
| 25-820 | 0.059 | 0.000 | 0.000 | 0.063 | 0.000 | 0.000 | 0.000 | 0.000 | 0.000 | 0.000 |
| 25-843 | 0.059 | 0.000 | 0.000 | 0.000 | 0.000 | 0.000 | 0.000 | 0.000 | 0.000 | 0.000 |
| 25-867 | 0.294 | 0.375 | 0.294 | 0.688 | 0.000 | 0.000 | 0.000 | 0.000 | 0.000 | 0.000 |
| 25-940 | 0.059 | 0.000 | 0.000 | 0.000 | 0.000 | 0.000 | 0.000 | 0.000 | 0.000 | 0.000 |
| 25-1002 | 0.059 | 0.000 | 0.000 | 0.000 | 0.000 | 0.000 | 0.000 | 0.000 | 0.000 | 0.000 |
| 25-1036 | 0.059 | 0.000 | 0.000 | 0.000 | 0.000 | 0.000 | 0.000 | 0.000 | 0.000 | 0.000 |
| 25-1086 | 0.059 | 0.000 | 0.000 | 0.063 | 0.000 | 0.000 | 0.000 | 0.000 | 0.000 | 0.000 |
| 25-1107 | 0.353 | 0.500 | 0.059 | 0.500 | 0.000 | 0.000 | 0.000 | 0.000 | 0.000 | 0.000 |
| 28-65 | 0.235 | 0.188 | 0.059 | 0.063 | 0.000 | 0.053 | 0.125 | 0.000 | 0.056 | 0.167 |
| 28-85 | 0.059 | 0.000 | 0.000 | 0.000 | 0.000 | 0.053 | 0.000 | 0.000 | 0.000 | 0.000 |
| 28-90 | 0.118 | 0.000 | 0.000 | 0.000 | 0.053 | 0.053 | 0.000 | 0.000 | 0.056 | 0.000 |
| 28-108 | 0.059 | 0.000 | 0.000 | 0.000 | 0.000 | 0.105 | 0.063 | 0.000 | 1.000 | 1.000 |
| 28-129 | 0.235 | 0.188 | 0.118 | 0.125 | 0.000 | 0.105 | 0.000 | 0.000 | 0.111 | 0.222 |
| 28-138 | 0.765 | 0.688 | 0.059 | 0.000 | 0.368 | 0.474 | 0.000 | 0.000 | 0.889 | 0.333 |
| 28-145 | 0.059 | 0.000 | 0.059 | 0.000 | 0.000 | 0.000 | 0.063 | 0.000 | 0.000 | 0.056 |
| 28-193 | 0.059 | 0.000 | 0.000 | 0.000 | 0.000 | 0.000 | 0.000 | 0.000 | 0.000 | 0.000 |
| 28-208 | 0.294 | 0.000 | 0.000 | 0.000 | 0.737 | 0.895 | 0.063 | 0.053 | 1.000 | 1.000 |
| 28-226 | 0.471 | 0.875 | 0.412 | 0.250 | 0.000 | 0.000 | 0.000 | 0.000 | 0.000 | 0.000 |
| 28-234 | 0.059 | 0.000 | 0.059 | 0.000 | 0.000 | 0.000 | 0.063 | 0.000 | 0.000 | 0.000 |
| 28-250 | 0.059 | 0.000 | 0.176 | 0.000 | 0.053 | 0.158 | 0.000 | 0.000 | 0.000 | 0.000 |
| 28-276 | 0.706 | 0.938 | 0.000 | 0.000 | 0.000 | 0.000 | 0.000 | 0.000 | 0.000 | 0.000 |
| 28-300 | 0.059 | 0.000 | 0.059 | 0.000 | 0.000 | 0.053 | 0.000 | 0.000 | 0.000 | 0.000 |
| 28-332 | 0.059 | 0.000 | 0.000 | 0.063 | 0.000 | 0.000 | 0.000 | 0.000 | 0.000 | 0.000 |
| 28-360 | 0.059 | 0.000 | 0.000 | 0.000 | 0.000 | 0.000 | 0.063 | 0.000 | 0.000 | 0.000 |
| 28-375 | 0.118 | 0.000 | 0.000 | 0.000 | 0.579 | 0.421 | 0.000 | 0.000 | 0.944 | 0.944 |
| 28-443 | 0.294 | 0.750 | 0.000 | 0.000 | 0.789 | 0.895 | 0.000 | 0.053 | 0.944 | 0.722 |
| 28-449 | 0.118 | 0.375 | 0.000 | 0.000 | 0.000 | 0.000 | 0.000 | 0.000 | 0.000 | 0.000 |
| 28-464 | 0.059 | 0.000 | 0.000 | 0.000 | 0.000 | 0.000 | 0.000 | 0.000 | 0.000 | 0.000 |
| 28-489 | 0.059 | 0.063 | 0.000 | 0.000 | 0.000 | 0.000 | 0.000 | 0.000 | 0.000 | 0.000 |
| 28-560 | 0.118 | 0.000 | 0.000 | 0.000 | 0.000 | 0.000 | 0.000 | 0.000 | 0.000 | 0.000 |
| 28-601 | 0.059 | 0.000 | 0.118 | 0.000 | 0.053 | 0.105 | 0.375 | 0.000 | 0.056 | 0.111 |
| 28-626 | 0.059 | 0.000 | 0.000 | 0.000 | 0.105 | 0.105 | 0.000 | 0.000 | 0.111 | 0.167 |
| 28-632 | 0.059 | 0.000 | 0.000 | 0.000 | 0.158 | 0.000 | 0.000 | 0.000 | 0.000 | 0.000 |
| 28-656 | 0.059 | 0.000 | 0.000 | 0.000 | 0.000 | 0.053 | 0.000 | 0.000 | 0.000 | 0.000 |
| 28-660 | 0.059 | 0.000 | 0.000 | 0.000 | 0.000 | 0.000 | 0.000 | 0.000 | 0.056 | 0.167 |
| 28-690 | 0.059 | 0.000 | 0.000 | 0.000 | 0.158 | 0.000 | 0.000 | 0.000 | 0.500 | 0.278 |
| 28-720 | 0.059 | 0.000 | 0.000 | 0.000 | 0.000 | 0.000 | 0.000 | 0.000 | 0.389 | 0.278 |
| 28-798 | 0.059 | 0.000 | 0.000 | 0.000 | 0.000 | 0.000 | 0.188 | 0.000 | 0.111 | 0.000 |
| 28-805 | 0.059 | 0.000 | 0.000 | 0.000 | 0.000 | 0.000 | 0.000 | 0.000 | 0.111 | 0.000 |
| 28-813 | 0.059 | 0.000 | 0.000 | 0.000 | 0.000 | 0.000 | 0.000 | 0.000 | 0.000 | 0.056 |
| 28-868 | 0.059 | 0.000 | 0.000 | 0.000 | 0.000 | 0.000 | 0.000 | 0.000 | 0.056 | 0.000 |
| 28-894 | 0.059 | 0.063 | 0.000 | 0.000 | 0.000 | 0.000 | 0.063 | 0.000 | 0.000 | 0.000 |
| 28-936 | 0.059 | 0.000 | 0.000 | 0.000 | 0.000 | 0.000 | 0.000 | 0.000 | 0.056 | 0.056 |
| 28-963 | 0.059 | 0.000 | 0.000 | 0.000 | 0.000 | 0.053 | 0.000 | 0.000 | 0.000 | 0.000 |
| 28-1005 | 0.059 | 0.000 | 0.000 | 0.000 | 0.000 | 0.000 | 0.000 | 0.000 | 0.056 | 0.056 |
| 28-1112 | 0.059 | 0.000 | 0.000 | 0.000 | 0.000 | 0.000 | 0.000 | 0.000 | 0.000 | 0.111 |
| 28-1182 | 0.059 | 0.000 | 0.000 | 0.000 | 0.000 | 0.000 | 0.000 | 0.000 | 0.056 | 0.000 |
| 31-63 | 0.059 | 0.000 | 0.000 | 0.000 | 0.000 | 0.000 | 0.000 | 0.000 | 0.000 | 0.000 |
| 31-80 | 0.118 | 0.000 | 0.000 | 0.000 | 0.000 | 0.000 | 0.000 | 0.000 | 0.000 | 0.000 |
| 31-90 | 0.118 | 0.000 | 0.000 | 0.000 | 0.000 | 0.000 | 0.000 | 0.000 | 0.000 | 0.000 |
| 31-123 | 0.059 | 0.000 | 0.000 | 0.000 | 0.211 | 0.105 | 0.375 | 0.000 | 0.000 | 0.000 |
| 31-132 | 0.176 | 0.000 | 0.000 | 0.000 | 0.000 | 0.000 | 0.000 | 0.000 | 0.000 | 0.000 |
| 31-156 | 0.118 | 0.000 | 0.000 | 0.000 | 0.000 | 0.000 | 0.000 | 0.000 | 0.000 | 0.000 |
| 31-166 | 0.176 | 0.000 | 0.000 | 0.000 | 0.053 | 0.105 | 0.063 | 0.053 | 0.000 | 0.000 |
| 31-172 | 0.118 | 0.000 | 0.000 | 0.000 | 0.263 | 0.158 | 0.125 | 0.211 | 0.000 | 0.056 |
| 31-177 | 0.059 | 0.000 | 0.000 | 0.000 | 0.053 | 0.000 | 0.000 | 0.000 | 0.000 | 0.000 |
| 31-186 | 0.118 | 0.000 | 0.000 | 0.000 | 0.053 | 0.053 | 0.313 | 0.316 | 0.000 | 0.000 |
| 31-192 | 0.176 | 0.000 | 0.000 | 0.000 | 0.158 | 0.105 | 0.063 | 0.000 | 0.000 | 0.000 |
| 31-199 | 0.059 | 0.000 | 0.000 | 0.000 | 0.263 | 0.000 | 0.125 | 0.053 | 0.000 | 0.000 |
| 31-218 | 0.059 | 0.000 | 0.000 | 0.000 | 0.000 | 0.000 | 0.063 | 0.000 | 0.000 | 0.000 |
| 31-223 | 0.941 | 0.938 | 1.000 | 1.000 | 0.895 | 0.947 | 0.875 | 0.632 | 0.500 | 0.222 |
| 31-240 | 0.118 | 0.000 | 0.000 | 0.000 | 0.000 | 0.053 | 0.000 | 0.053 | 0.000 | 0.000 |
| 31-249 | 0.059 | 0.000 | 0.000 | 0.000 | 0.000 | 0.000 | 0.063 | 0.000 | 0.000 | 0.000 |
| 31-258 | 0.059 | 0.000 | 0.000 | 0.000 | 0.000 | 0.000 | 0.000 | 0.000 | 0.000 | 0.000 |
| 31-265 | 0.118 | 0.063 | 0.059 | 0.063 | 0.053 | 0.053 | 0.188 | 0.053 | 0.000 | 0.000 |
| 31-278 | 0.294 | 0.000 | 0.000 | 0.000 | 0.895 | 0.895 | 0.813 | 0.789 | 0.944 | 0.500 |
| 31-290 | 0.059 | 0.000 | 0.000 | 0.000 | 0.000 | 0.000 | 0.000 | 0.000 | 0.000 | 0.000 |
| 31-297 | 0.059 | 0.000 | 0.000 | 0.000 | 0.000 | 0.000 | 0.125 | 0.000 | 0.000 | 0.000 |
| 31-302 | 0.118 | 0.000 | 0.059 | 0.000 | 0.263 | 0.158 | 0.625 | 0.368 | 0.000 | 0.000 |
| 31-310 | 0.059 | 0.000 | 0.000 | 0.000 | 0.000 | 0.053 | 0.000 | 0.000 | 0.000 | 0.000 |
| 31-320 | 0.059 | 0.000 | 0.000 | 0.000 | 0.105 | 0.053 | 0.438 | 0.053 | 0.000 | 0.000 |
| 31-336 | 0.118 | 0.000 | 0.000 | 0.000 | 0.000 | 0.000 | 0.188 | 0.000 | 0.000 | 0.000 |
| 31-381 | 0.059 | 0.000 | 0.000 | 0.000 | 0.053 | 0.000 | 0.313 | 0.053 | 0.056 | 0.000 |
| 31-389 | 0.118 | 0.000 | 0.000 | 0.000 | 0.263 | 0.158 | 0.125 | 0.000 | 0.278 | 0.222 |
| 31-398 | 0.059 | 0.000 | 0.000 | 0.000 | 0.053 | 0.000 | 0.250 | 0.105 | 0.111 | 0.000 |
| 31-403 | 0.059 | 0.000 | 0.000 | 0.000 | 0.158 | 0.316 | 0.313 | 0.053 | 0.167 | 0.000 |
| 31-460 | 0.059 | 0.063 | 0.000 | 0.000 | 0.000 | 0.000 | 0.000 | 0.000 | 0.000 | 0.000 |
| 31-509 | 0.059 | 0.000 | 0.000 | 0.000 | 0.000 | 0.000 | 0.000 | 0.000 | 0.000 | 0.000 |
| 31-520 | 0.059 | 0.063 | 0.000 | 0.000 | 0.053 | 0.000 | 0.188 | 0.000 | 0.444 | 0.444 |
| 31-567 | 0.176 | 0.000 | 0.000 | 0.000 | 0.000 | 0.000 | 0.000 | 0.000 | 0.000 | 0.000 |
| 31-609 | 0.059 | 0.000 | 0.000 | 0.063 | 0.053 | 0.000 | 0.000 | 0.000 | 0.000 | 0.000 |
| 31-620 | 0.059 | 0.000 | 0.000 | 0.000 | 0.053 | 0.000 | 0.000 | 0.000 | 0.000 | 0.000 |
| 31-647 | 0.059 | 0.000 | 0.000 | 0.000 | 0.000 | 0.000 | 0.000 | 0.000 | 0.056 | 0.000 |
| 31-654 | 0.118 | 0.000 | 0.000 | 0.000 | 0.000 | 0.000 | 0.063 | 0.000 | 0.889 | 0.500 |
| 31-669 | 0.118 | 0.000 | 0.000 | 0.000 | 0.158 | 0.158 | 0.063 | 0.000 | 0.833 | 0.556 |
| 31-709 | 0.647 | 0.750 | 0.118 | 0.625 | 0.947 | 1.000 | 0.938 | 0.842 | 1.000 | 0.944 |
| 31-769 | 0.059 | 0.000 | 0.000 | 0.000 | 0.000 | 0.000 | 0.000 | 0.000 | 0.000 | 0.000 |
| 31-860 | 0.118 | 0.000 | 0.000 | 0.000 | 0.000 | 0.000 | 0.000 | 0.000 | 0.000 | 0.000 |
| 31-970 | 0.059 | 0.000 | 0.000 | 0.000 | 0.000 | 0.000 | 0.000 | 0.000 | 0.000 | 0.000 |
| 31-1075 | 0.059 | 0.063 | 0.000 | 0.000 | 0.000 | 0.000 | 0.000 | 0.000 | 0.000 | 0.000 |
| 31-1150 | 0.059 | 0.000 | 0.000 | 0.000 | 0.000 | 0.000 | 0.000 | 0.053 | 0.000 | 0.000 |
| 32-73 | 0.059 | 0.000 | 0.118 | 0.000 | 0.000 | 0.000 | 0.125 | 0.053 | 0.056 | 0.000 |
| 32-85 | 0.706 | 0.938 | 0.824 | 0.875 | 0.421 | 0.895 | 0.750 | 0.579 | 0.000 | 0.278 |
| 32-95 | 1.000 | 0.938 | 0.882 | 0.938 | 0.895 | 0.947 | 0.875 | 0.842 | 1.000 | 1.000 |
| 32-109 | 0.059 | 0.000 | 0.000 | 0.000 | 0.000 | 0.000 | 0.000 | 0.053 | 0.000 | 0.000 |
| 32-115 | 0.059 | 0.000 | 0.059 | 0.000 | 0.000 | 0.000 | 0.063 | 0.000 | 0.000 | 0.000 |
| 32-145 | 0.765 | 0.938 | 0.882 | 0.938 | 0.789 | 0.947 | 0.750 | 0.526 | 1.000 | 0.833 |
| 32-161 | 0.235 | 0.000 | 0.000 | 0.000 | 0.000 | 0.000 | 0.063 | 0.000 | 0.000 | 0.000 |
| 32-166 | 0.059 | 0.000 | 0.000 | 0.000 | 0.053 | 0.000 | 0.000 | 0.000 | 0.000 | 0.000 |
| 32-183 | 0.235 | 0.063 | 0.941 | 0.875 | 0.316 | 0.316 | 0.188 | 0.053 | 0.167 | 0.056 |
| 32-211 | 0.882 | 0.875 | 0.882 | 0.938 | 0.579 | 0.737 | 0.813 | 0.842 | 0.944 | 0.722 |
| 32-238 | 0.529 | 0.813 | 1.000 | 0.938 | 0.684 | 0.632 | 0.875 | 0.684 | 0.778 | 0.611 |
| 32-250 | 0.059 | 0.000 | 0.059 | 0.000 | 0.000 | 0.000 | 0.000 | 0.000 | 0.000 | 0.000 |
| 32-258 | 0.059 | 0.000 | 0.059 | 0.000 | 0.000 | 0.000 | 0.063 | 0.000 | 0.000 | 0.000 |
| 32-318 | 0.471 | 0.000 | 0.000 | 0.000 | 0.000 | 0.000 | 0.000 | 0.000 | 0.000 | 0.000 |
| 32-327 | 0.765 | 0.938 | 0.941 | 0.938 | 0.842 | 0.947 | 0.750 | 0.842 | 1.000 | 0.944 |
| 32-344 | 0.059 | 0.000 | 0.000 | 0.000 | 0.000 | 0.000 | 0.063 | 0.000 | 0.000 | 0.000 |
| 32-349 | 0.059 | 0.000 | 0.059 | 0.000 | 0.053 | 0.000 | 0.000 | 0.000 | 0.000 | 0.000 |
| 32-357 | 0.059 | 0.000 | 0.000 | 0.000 | 0.000 | 0.000 | 0.000 | 0.000 | 0.056 | 0.000 |
| 32-417 | 0.059 | 0.000 | 0.882 | 0.875 | 0.000 | 0.053 | 0.125 | 0.000 | 0.389 | 0.278 |
| 32-427 | 0.059 | 0.000 | 0.059 | 0.000 | 0.000 | 0.000 | 0.000 | 0.000 | 0.000 | 0.000 |
| 32-438 | 0.059 | 0.000 | 0.059 | 0.000 | 0.000 | 0.000 | 0.000 | 0.000 | 0.000 | 0.000 |
| 32-460 | 0.059 | 0.000 | 0.000 | 0.000 | 0.000 | 0.000 | 0.000 | 0.053 | 0.000 | 0.000 |
| 32-475 | 0.118 | 0.000 | 0.941 | 0.875 | 0.053 | 0.105 | 0.313 | 0.053 | 0.389 | 0.056 |
| 32-497 | 0.059 | 0.000 | 0.059 | 0.000 | 0.000 | 0.000 | 0.000 | 0.000 | 0.000 | 0.000 |
| 32-512 | 0.059 | 0.000 | 0.059 | 0.000 | 0.053 | 0.000 | 0.000 | 0.000 | 0.000 | 0.000 |
| 32-539 | 0.059 | 0.000 | 0.000 | 0.000 | 0.000 | 0.000 | 0.063 | 0.000 | 0.000 | 0.000 |
| 32-605 | 0.059 | 0.000 | 0.059 | 0.000 | 0.053 | 0.000 | 0.000 | 0.000 | 0.000 | 0.000 |
| 32-698 | 0.059 | 0.000 | 0.059 | 0.000 | 0.000 | 0.000 | 0.000 | 0.000 | 0.000 | 0.000 |
| 32-708 | 0.059 | 0.000 | 0.000 | 0.000 | 0.000 | 0.000 | 0.000 | 0.000 | 0.056 | 0.000 |
| 32-1008 | 0.059 | 0.000 | 0.000 | 0.063 | 0.000 | 0.000 | 0.000 | 0.000 | 0.000 | 0.000 |
| 32-1033 | 0.059 | 0.000 | 0.000 | 0.063 | 0.000 | 0.000 | 0.000 | 0.000 | 0.000 | 0.000 |
| 32-1041 | 0.059 | 0.000 | 0.000 | 0.063 | 0.000 | 0.000 | 0.000 | 0.000 | 0.000 | 0.000 |
| 32-1123 | 0.059 | 0.000 | 0.000 | 0.000 | 0.000 | 0.000 | 0.000 | 0.000 | 0.000 | 0.056 |
| 36-63 | 0.118 | 0.000 | 0.059 | 0.000 | 0.053 | 0.000 | 0.000 | 0.000 | 0.000 | 0.056 |
| 36-72 | 0.059 | 0.000 | 0.059 | 0.000 | 0.000 | 0.000 | 0.063 | 0.000 | 0.000 | 0.000 |
| 36-78 | 0.059 | 0.000 | 0.000 | 0.000 | 0.053 | 0.000 | 0.000 | 0.000 | 0.000 | 0.000 |
| 36-85 | 0.118 | 0.250 | 0.176 | 0.063 | 0.053 | 0.053 | 0.000 | 0.158 | 0.167 | 0.222 |
| 36-95 | 0.412 | 0.375 | 0.765 | 0.500 | 0.105 | 0.421 | 0.125 | 0.211 | 0.667 | 0.556 |
| 36-106 | 0.059 | 0.000 | 0.000 | 0.000 | 0.053 | 0.000 | 0.000 | 0.053 | 0.000 | 0.000 |
| 36-111 | 0.059 | 0.000 | 0.059 | 0.000 | 0.000 | 0.000 | 0.000 | 0.053 | 0.000 | 0.000 |
| 36-122 | 0.118 | 0.000 | 0.000 | 0.000 | 0.000 | 0.000 | 0.000 | 0.000 | 0.000 | 0.056 |
| 36-135 | 0.235 | 0.125 | 0.235 | 0.250 | 0.000 | 0.000 | 0.500 | 0.000 | 0.167 | 0.056 |
| 36-143 | 0.235 | 0.000 | 0.000 | 0.000 | 0.000 | 0.053 | 0.000 | 0.000 | 0.111 | 0.000 |
| 36-148 | 0.529 | 0.125 | 0.588 | 0.750 | 0.000 | 0.000 | 0.500 | 0.053 | 0.056 | 0.000 |
| 36-161 | 0.059 | 0.125 | 0.059 | 0.125 | 0.000 | 0.000 | 0.250 | 0.000 | 0.056 | 0.000 |
| 36-187 | 0.294 | 0.000 | 0.000 | 0.000 | 0.000 | 0.053 | 0.063 | 0.053 | 0.000 | 0.000 |
| 36-217 | 0.412 | 0.000 | 0.353 | 0.250 | 0.316 | 0.105 | 0.063 | 0.316 | 0.000 | 0.000 |
| 36-231 | 0.294 | 0.063 | 0.000 | 0.000 | 0.105 | 0.211 | 0.000 | 0.000 | 0.000 | 0.000 |
| 36-238 | 0.471 | 0.125 | 0.706 | 0.375 | 0.158 | 0.053 | 0.250 | 0.053 | 0.056 | 0.000 |
| 36-256 | 0.059 | 0.000 | 0.118 | 0.000 | 0.000 | 0.000 | 0.000 | 0.000 | 0.000 | 0.000 |
| 36-263 | 0.176 | 0.000 | 0.000 | 0.063 | 0.053 | 0.000 | 0.063 | 0.000 | 0.000 | 0.000 |
| 36-276 | 0.235 | 0.125 | 0.765 | 0.313 | 0.105 | 0.105 | 0.688 | 0.000 | 0.056 | 0.000 |
| 36-293 | 0.294 | 0.000 | 0.059 | 0.000 | 0.421 | 0.368 | 0.125 | 0.474 | 0.000 | 0.000 |
| 36-359 | 0.176 | 0.000 | 0.059 | 0.000 | 0.000 | 0.000 | 0.000 | 0.000 | 0.000 | 0.000 |
| 36-368 | 0.176 | 0.000 | 0.235 | 0.375 | 0.368 | 0.421 | 0.063 | 0.421 | 0.000 | 0.000 |
| 36-381 | 0.353 | 0.063 | 0.059 | 0.063 | 0.316 | 0.211 | 0.188 | 0.211 | 0.000 | 0.000 |
| 36-424 | 0.059 | 0.000 | 0.000 | 0.000 | 0.158 | 0.105 | 0.000 | 0.053 | 0.000 | 0.000 |
| 36-460 | 0.059 | 0.000 | 0.000 | 0.000 | 0.000 | 0.000 | 0.063 | 0.053 | 0.000 | 0.000 |
| 36-535 | 0.118 | 0.313 | 0.765 | 0.563 | 0.000 | 0.000 | 0.625 | 0.000 | 0.056 | 0.000 |
| 36-555 | 0.059 | 0.000 | 0.118 | 0.000 | 0.000 | 0.000 | 0.000 | 0.000 | 0.000 | 0.000 |
| 36-640 | 0.059 | 0.000 | 0.000 | 0.000 | 0.000 | 0.000 | 0.000 | 0.000 | 0.111 | 0.000 |
| 36-678 | 0.059 | 0.000 | 0.000 | 0.000 | 0.421 | 0.421 | 0.000 | 0.316 | 0.000 | 0.000 |
| 36-696 | 0.059 | 0.000 | 0.000 | 0.000 | 0.368 | 0.368 | 0.000 | 0.105 | 0.000 | 0.000 |
| 36-780 | 0.059 | 0.250 | 0.000 | 0.375 | 0.000 | 0.000 | 0.063 | 0.000 | 0.056 | 0.000 |
| 36-793 | 0.059 | 0.250 | 0.000 | 0.188 | 0.000 | 0.000 | 0.000 | 0.000 | 0.056 | 0.000 |
| 36-815 | 0.059 | 0.000 | 0.059 | 0.000 | 0.000 | 0.000 | 0.000 | 0.000 | 0.000 | 0.000 |
| 36-913 | 0.059 | 0.000 | 0.059 | 0.000 | 0.000 | 0.000 | 0.000 | 0.000 | 0.000 | 0.000 |
| 36-921 | 0.059 | 0.000 | 0.059 | 0.000 | 0.000 | 0.000 | 0.000 | 0.000 | 0.000 | 0.000 |
| 36-992 | 0.059 | 0.000 | 0.000 | 0.063 | 0.000 | 0.000 | 0.000 | 0.000 | 0.000 | 0.000 |
| 36-1002 | 0.059 | 0.000 | 0.000 | 0.125 | 0.000 | 0.000 | 0.000 | 0.000 | 0.000 | 0.000 |
| 36-1070 | 0.059 | 0.000 | 0.000 | 0.063 | 0.000 | 0.000 | 0.063 | 0.000 | 0.000 | 0.000 |
| 36-1081 | 0.059 | 0.000 | 0.000 | 0.063 | 0.000 | 0.000 | 0.063 | 0.000 | 0.000 | 0.000 |
| 36-1107 | 0.059 | 0.000 | 0.000 | 0.188 | 0.000 | 0.000 | 0.000 | 0.000 | 0.000 | 0.000 |
| 36-1121 | 0.118 | 0.000 | 0.000 | 0.000 | 0.000 | 0.000 | 0.000 | 0.000 | 0.000 | 0.000 |
