# Supplementary material for: Landscape genetics reveals that adaptive genetic divergence in Pinus bungeana (Pinaceae) is driven by environmental variables relating to ecological habitats
Source: BMC Evol Biol. 2019 Aug 1;19:160. doi: 10.1186/s12862-019-1489-x (PMC6676527; doi:10.1186/s12862-019-1489-x)
Supplement: Supplementary file 3 — Environmental variables for each location from the WorldClim database. (DOCX 16 kb) [file 12862_2019_1489_MOESM3_ESM.docx]

**Additional file 3** Environmental variables for each location from the WorldClim database.

| Population no. and code | Environmental variables | | | | | | | | | | | | | | | | | | |
| --- | --- | --- | --- | --- | --- | --- | --- | --- | --- | --- | --- | --- | --- | --- | --- | --- | --- | --- | --- |
|  | Bio1 | Bio2 | Bio3 | Bio4 | Bio5 | Bio6 | Bio7 | Bio8 | Bio9 | Bio10 | Bio11 | Bio12 | Bio13 | Bio14 | Bio15 | Bio16 | Bio17 | Bio18 | Bio19 |
| 1.SXNN | 9.0 | 12.7 | 31.1 | 1030.5 | 27.9 | -12.9 | 40.8 | 19.5 | -4.8 | 21.1 | -4.8 | 646 | 174 | 6 | 103.0 | 398 | 20 | 393 | 20 |
| 2.GSLG | 11.4 | 8.9 | 27.4 | 853.6 | 27.7 | -4.9 | 32.6 | 20.5 | 0.4 | 21.8 | 0.4 | 704 | 126 | 5 | 82.3 | 377 | 18 | 339 | 18 |
| 3.SXLJ | 10.1 | 9.0 | 26.1 | 924.3 | 26.6 | -7.7 | 34.3 | 20.0 | -1.7 | 21.4 | -1.7 | 734 | 144 | 9 | 73.2 | 368 | 31 | 337 | 31 |
| 4.SXWZ | 14.0 | 8.8 | 27.7 | 853.1 | 29.8 | -2 | 31.8 | 23.3 | 3.2 | 24.4 | 3.2 | 891 | 164 | 5 | 78.6 | 461 | 22 | 393 | 22 |
| 5.SXWJ | 7.7 | 13.0 | 30.9 | 1074.6 | 27.2 | -14.9 | 42.1 | 18.7 | -6.3 | 20.3 | -6.3 | 415 | 125 | 2 | 114.1 | 273 | 9 | 265 | 9 |
| 6.HNSN | 11.2 | 11.4 | 30.1 | 988.3 | 28.9 | -8.8 | 37.7 | 21.4 | -1.8 | 23.0 | -1.8 | 659 | 157 | 8 | 87.2 | 369 | 28 | 358 | 28 |
| 7.SXWL | 10.1 | 9.4 | 26.5 | 963.6 | 27 | -8.6 | 35.6 | 20.3 | -2.3 | 21.8 | -2.3 | 631 | 128 | 8 | 77.4 | 329 | 26 | 304 | 26 |
| 8.GSMJ | 9.0 | 9.3 | 28.2 | 850.0 | 25.4 | -7.6 | 33 | 18.1 | -2.0 | 19.2 | -2.0 | 649 | 120 | 4 | 83.0 | 346 | 15 | 315 | 15 |
| 9.SCGP | 14.3 | 8.8 | 30.4 | 726.9 | 28.5 | -0.6 | 29.1 | 22.1 | 4.8 | 23.0 | 4.8 | 868 | 195 | 5 | 96.2 | 515 | 18 | 494 | 18 |
| 10.HBLJ | 13.7 | 9.3 | 28.2 | 876.7 | 29.6 | -3.3 | 32.9 | 23.4 | 2.6 | 24.4 | 2.6 | 972 | 175 | 20 | 64.4 | 449 | 71 | 440 | 71 |
